# Heat shock transcription factors demonstrate a distinct mode of interaction with mitotic chromosomes

**DOI:** 10.1093/nar/gkad304

**Published:** 2023-04-28

**Authors:** Rachel M Price, Marek A Budzyński, Junzhou Shen, Jennifer E Mitchell, James Z J Kwan, Sheila S Teves

**Affiliations:** Department of Biochemistry and Molecular Biology, Life Sciences Institute, University of British Columbia, 2350 Health Sciences Mall, Vancouver BC V6T 1Z3, Canada; Department of Biochemistry and Molecular Biology, Life Sciences Institute, University of British Columbia, 2350 Health Sciences Mall, Vancouver BC V6T 1Z3, Canada; Department of Biochemistry and Molecular Biology, Life Sciences Institute, University of British Columbia, 2350 Health Sciences Mall, Vancouver BC V6T 1Z3, Canada; Department of Biochemistry and Molecular Biology, Life Sciences Institute, University of British Columbia, 2350 Health Sciences Mall, Vancouver BC V6T 1Z3, Canada; Department of Biochemistry and Molecular Biology, Life Sciences Institute, University of British Columbia, 2350 Health Sciences Mall, Vancouver BC V6T 1Z3, Canada; Department of Biochemistry and Molecular Biology, Life Sciences Institute, University of British Columbia, 2350 Health Sciences Mall, Vancouver BC V6T 1Z3, Canada

## Abstract

A large number of transcription factors have been shown to bind and interact with mitotic chromosomes, which may promote the efficient reactivation of transcriptional programs following cell division. Although the DNA-binding domain (DBD) contributes strongly to TF behavior, the mitotic behaviors of TFs from the same DBD family may vary. To define the mechanisms governing TF behavior during mitosis in mouse embryonic stem cells, we examined two related TFs: Heat Shock Factor 1 and 2 (HSF1 and HSF2). We found that HSF2 maintains site-specific binding genome-wide during mitosis, whereas HSF1 binding is somewhat decreased. Surprisingly, live-cell imaging shows that both factors appear excluded from mitotic chromosomes to the same degree, and are similarly more dynamic in mitosis than in interphase. Exclusion from mitotic DNA is not due to extrinsic factors like nuclear import and export mechanisms. Rather, we found that the HSF DBDs can coat mitotic chromosomes, and that HSF2 DBD is able to establish site-specific binding. These data further confirm that site-specific binding and chromosome coating are independent properties, and that for some TFs, mitotic behavior is largely determined by the non-DBD regions.

## Graphical Abstract

**Graphical Abstract:**
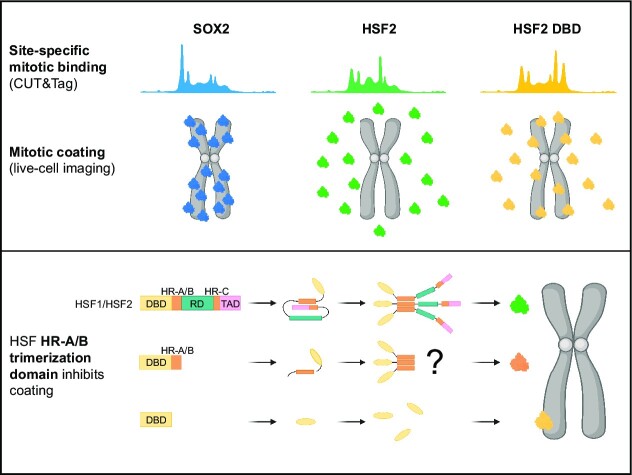
Interactions between transcription factors and mitotic chromosome can be classified as site-specific binding and mitotic coating. We found that, for HSF2, these two behaviors are mediated by distinct domains within HSF2. DBD - DNA binding domain; HR-A/B - heptad repeats A and B; RD - regulatory domain; TAD - transactivation domain.

## INTRODUCTION

Mitosis is accompanied by major changes in cellular morphology and function. At the onset of M phase, DNA is globally condensed and reorganized to form characteristic mitotic chromosomes (1), and transcription from mitotic chromosomes is largely shut down (2). The nuclear membrane also loses its continuity in mitotic cells and no longer serves as a tight barrier between the cytoplasm and chromatin (3). Given that transcription is rapidly re-established after cell division (2,4), mechanisms must exist to facilitate the precise and accurate reestablishment of gene expression after mitosis (5). One proposed mechanism, termed mitotic bookmarking, suggests that the binding of proteins, mainly transcription factors (TFs), to specific genomic loci during mitosis marks genes for rapid activation after cell division (6).

Early studies using chromatin immunoprecipitation (ChIP)-based methods identified only a few TFs that bind to specific loci during mitosis, including HSF2, TFIID and TFIIB (7,8). Immunofluorescence analysis showed that most TFs are displaced from mitotic chromatin, further supporting the hypothesis that mitotic bookmarking is rare (9). This rarity was due in part to formaldehyde fixation, which was found to artificially evict proteins from DNA during mitosis (10–12). Indeed, a recent study using ChIP-based methods with alternative fixatives detected more site-specific binding during mitosis by various TFs (12), and advances in live-cell imaging led to the identification of an increasing number of TFs that are enriched on mitotic DNA, including GATA1, FOXA1, SOX2, TBP, CTCF, KLF4 and ESRRB (10,13–18). Since then, studies using imaging and proteomics approaches have identified hundreds of proteins, including TFs, that interact with DNA during mitosis (19–21). A large screen of TF localization during mitosis by live-cell imaging reported varying degrees of DNA-TF interactions, from strongly enriched on mitotic DNA, through moderately enriched, to clearly excluded (19). These studies show that mitotic DNA-TF interactions are best measured by live-cell imaging and/or by chromatin-binding assays that avoid formaldehyde fixation, with the assumption that the chromatin association measured by one method corresponds with the association measured by the other. However, chromatin-binding assays measure site-specific binding of TFs during mitosis, whereas live-cell imaging techniques visualize global mitotic chromatin ‘coating’, which is heavily influenced by non-specific interactions between the TF and the DNA (12,16). Indeed, some proteins that are unable to bind DNA site-specifically still demonstrate global mitotic coating as visualized by live imaging (10,12,16). Although coating is likely a function of both site-specific binding and non-specific interactions (10,16), live-cell imaging cannot provide information about TF binding at specific loci.

Regardless of the method of measurement, certain TF domains are critical for DNA–TF interaction during mitosis. The DNA-binding domain (DBD) has been shown to drive much of TF behavior (22–24), and would be expected to play a major role in mitotic DNA-TF interactions as well. Indeed, previous work by Raccaud et al. suggests that the type of DBD predicts the strength of DNA-TF interactions to a certain degree (19). Additionally, the DBD is necessary for mitotic bookmarking in some cases (10). Yet, TFs with highly similar DBDs often interact differently with mitotic DNA (19,25), and the mechanisms that drive TF interactions with mitotic DNA remain largely unknown. Even less understood than the DBD is the role of the nuclear import mechanism and the nuclear localization signal (NLS) in mitotic DNA-TF interactions (10,19,26). Both the NLS and active nuclear import have been suggested to promote mitotic DNA–protein association. Mutating the NLS of SOX2 resulted in eviction of the TF from mitotic DNA in mESCs, while adding the simian virus 40 (SV40) NLS—a strong viral nuclear localization signal (27)—to HaloTag alone promoted coating of the mitotic DNA (10). However, other work suggested that addition of positively charged amino acids was sufficient to localize proteins to mitotic chromosomes, with or without active nuclear import (19). Additionally, importin-β was shown to be involved in the retention of HNF1β on mitotic DNA in MDCK cells, indicating a link between active nuclear import and mitotic DNA-TF interactions (26).

An example of a pair of TFs with highly conserved DBDs that are shown to have distinct mitotic behaviors is Heat Shock Factors 1 and 2 (HSF1 and HSF2) (Supplementary Figure S1A) (28). Known as the primary regulators of the evolutionarily conserved Heat Shock Response defense mechanism, activated HSFs trimerize and drive the expression of genes in response to protein-damaging stress (29). In vertebrates, HSF1 and HSF2 are ubiquitously expressed in all tissues (30), and have been shown to form homo- and hetero-trimers upon activation (31). HSF2 was one of the earliest TFs identified to bind to mitotic DNA at specific loci, as measured by ChIP (7,25). In contrast, eviction of HSF1 from mitotic chromatin was established by multiple studies including those that used live-cell imaging approaches (9,10,25).

Using a combination of genomics and live-cell imaging in mESCs, we investigate the molecular underpinnings behind the interactions of HSFs with mitotic DNA. Using Cleavage Under Targets and Tagmentation (CUT&Tag) (32), we found that both endogenous HSF1 and HSF2 bind site-specifically to mitotic DNA, although at different levels, but live-cell imaging shows that both HaloTagged HSF1 and HSF2 are evicted from mitotic chromatin to the same degree. Despite differing levels of site-specific binding, the dynamics of HSF1-Halo and Halo-HSF2 are remarkably similar when examined with single-molecule live-cell imaging and single-particle tracking (SPT). Additionally, we find that the SV40 NLS is unable to induce mitotic coating of Halo-HSF2, though it does so for HaloTag alone. However, the truncated HSF DBDs demonstrate increased enrichment on mitotic DNA, and this coating is inhibited by the heptad repeats A and B (HR-A/B) within each HSF. Though able to coat in isolation, neither DBD is able to induce coating of the non-DBD domains of the complementary HSF. Therefore, we demonstrate that the non-DBD regions of HSF1 and HSF2 dictate their exclusion from mitotic DNA, in contrast to other TFs such as SOX2 and SOX13 whose enrichment is driven almost entirely by the DBD.

## MATERIALS AND METHODS

### Cell culture

Mouse ES cells (JM8.N4, RRID: CVCL_J962) were used for all experiments and obtained as previously described (15). ES cells were cultured on 0.1% gelatin-coated plates in ESC DMEM (Corning 10101CV, contains 4.5 g/l glucose, 110 mg/l sodium pyruvate, glutagro, and 15 mg/l phenol red) with 15% FBS (HyClone), 0.1 mM MEM non-essential amino acids (Gibco), 2 mM l-glutamine (Gibco), 0.1 mM 2-mercaptoethanol (Sigma), 1× Penicillin Streptomycin solution (Corning), and 1000 units/ml of ESGRO LIF (Chem-icon). ESCs were fed daily, cultured at 37°C in a 5% CO_2_ incubator, and passaged every 2 days by trypsinization. For Leptomycin B treatment, cells were treated with 10 ng/ml Leptomycin B (Sigma) at 37°C in a 5% CO_2_ incubator for 1 hour. For all imaging experiments, cells were imaged in DMEM without phenol red (Gibco 31053028, contains 4.5 g/l glucose) with 110 mg/l sodium pyruvate (Gibco) and all other components previously listed.

### Mitotic synchronization

Mouse ESCs were treated with 50 ng/ml nocodazole (Sigma) at 37°C in a 5% CO_2_ incubator for 6 h. After nocodazole removal, cells were washed with 1× PBS and mitotic cells were collected via shake-off. Cells were fixed in 70% ethanol and purity of the mitotic fraction (mitotic index) was monitored by flow cytometry and cell counting. For flow cytometry, asynchronous and shake-off cells were stained with propidium iodide (20 μg/ml; Sigma-Aldrich) and analyzed on the FACS Canto system (BD Bioscience). The flow cytometry profiles were analyzed using FlowJo software. Live cells were gated and the histogram function was plotted. For cell counting, cells were permeabilized with 0.1% Triton-X, stained with 300 nM DAPI (Invitrogen), and images were acquired using a microscope (Leica DMI6000 B). Mitotic cells were identified based on the presence and morphology of condensed mitotic chromosomes and quantified as percent of total cells within the image field.

### Cloning

To generate N-terminal HaloTagged TFs, the coding sequence of each TF was cloned into a Piggybac vector containing either a 3X-Flag-Halo-TEV or HA-3X-Flag-Halo-TEV construct upstream of a multiple cloning site. For C-terminal HaloTagged TFs, the coding sequence of each TF was cloned into a Piggybac vector containing either a TEV-Halo-1X-Flag or TEV-Halo-1X-Flag-HA construct downstream of a multiple cloning site. All primers used for cloning are listed in Supplementary Table S1. All HaloTagged TF plasmids are deposited on Addgene.

### Transfection

Mouse ES cells were grown to ∼50% confluency on 0.1% gelatin-coated 6-well plates at 37°C in a 5% CO_2_ incubator. Stably expressing cell lines were generated by transfecting 1 μg of the HaloTagged TF Piggybac construct together with 1 μg of the Super Piggybac transposase plasmid (gifted from the Tjian Lab) using Lipofectamine 2000 (Invitrogen) according to the manufacturer's instructions. After 24 h, 500 μg/ml G418 (Fisher) was added. Cell media containing antibiotics was refreshed daily until all negative control cells were dead. A similar method was used to stably express H2B-GFP under 0.6 μg/ml Puromycin (ThermoFisher).

### Generation of *hsf1* and *hsf2* knock-out cells with CRISPR-cas9

Two sets of guide RNAs (gRNAs) targeting the entire locus of *Hsf1* or *Hsf2* respectively were designed using Benchling CRISPR Guide RNA Design tool (https://www.benchling.com/crispr) and cloned into the px458 gRNA expression plasmid. Mouse ES cells were transfected with 1 μg of plasmid containing Cas9 and gRNAs using Lipofectamine 2000 (Invitrogen) according to the manufacturer's instructions. Cells were sorted two days after transfection, and 20 000 transfected-positive cells were plated onto a 15 cm tissue culture plate. One week after plating, individual colonies were picked up and trypsinized. 80% of each cell colony was used for genotyping, while 20% of each cell colony was grown for maintenance. For genotyping, cells were lysed using DirectPCR lysis reagent (Viagen Biotech) according to the manufacturer protocol and lysates were used in a screening PCR to identify edited cells. Identified clones were further validated using Sanger sequencing and Western blotting. A list of gRNAs and primers used for gene editing are in Supplementary Table S2.

### Live-cell imaging

Mouse ES cells were grown on 0.1% gelatin-coated chambered coverslips (Ibidi, μ-Slide 4 Well 80426 or 2 Well 80286) at 37°C in a 5% CO_2_ incubator for 24 h. Cells were labeled with 200 nM JF549 (Lavis Lab) for 30 min at 37°C in a 5% CO_2_ incubator and washed 3 times with 1× PBS for 5 min each. Cells were imaged with a Leica SP8 confocal microscope at 37°C in ESC DMEM without phenol red. Chromatin enrichment quantification was performed with ImageJ as follows: the mean HaloTag fluorescence intensity was calculated in the area of the mitotic chromatin and across the whole mitotic cell. The chromatin enrichment score was calculated by taking log_2_ of the chromatin mean divided by the whole cell mean.

### Western blot

Mouse ES cells were cultured to ∼90% confluency on 0.1% gelatin-coated plates at 37°C in a 5% CO_2_ incubator. After indicated treatments, cells were washed with 1× PBS, trypsinized, and pelleted by centrifuging at 600 g. Cell pellets were lysed in lysis buffer (150 mM NaCl, 50 mM HEPES pH 7.6, 1 mM EDTA, 2 mM MgCl_2_, 1% Triton X, 10% Glycerol, 1× Roche cOmplete EDTA-free Protease Inhibitor, 5 mM PMSF) for 15 min on ice. Lysates were pelleted at 4°C for 15 min at 16 000 g. The supernatants were saved, mixed with 4× Laemmli buffer, and boiled at 95°C for 5 min. Proteins were separated via 7, 8, 9 or 10% SDS-PAGE and wet transferred to Nitrocellulose membranes (VWR, CA10061-084) at 4°C. Membranes were blocked with blocking buffer [1× PBS, 0.1% Tween (Fisher)] containing either 5% non-fat milk powder or 3% BSA (Sigma-Aldrich) at room temperature for 40 min. Membranes were incubated in α-HSF1 1:1000 (Abcam, ab2923), α-HSF2 1:1000 (33), α-H3K27me3 1:7000 (Cell Signaling Technologies, C36B11), α-Tubulin 1:7000 (Abcam, ab6046), α-SOX2 1:1000 (Cedarline, 39844), α-HaloTag 1:1000 (Promega, G9211), or α-Flag 1:5000 (Sigma-Aldrich, F3165) overnight at 4°C. Membranes were washed in 1× PBS with 0.1% Tween 3 times for 5 min each at room temperature. Membranes were then incubated in IRDye 800CW Goat anti-mouse (Cedarlane, 926-32210) or IRDye 800CW Goat anti-rabbit (Cedarlane, 925–32211) secondary antibodies (1:20000) at room temperature for 45 min. Finally, membranes were washed in 1× PBS with 0.1% Tween 3 times for 5 min each before scanning using the ChemiDoc™ MP Imaging System (Bio-Rad). Note: membranes blotted with α-HSF2 were boiled for 10 min prior to blocking (34).

### Single particle tracking: slow tracking

Cells were grown on gelatin-coated 35mm glass-bottomed dishes (Ibidi, μ-Dish 35 mm, high Glass Bottom 81158) at 37°C in a 5% CO_2_ incubator for 24 h. Cells were labeled with 25 pM (H2B-Halo control) or 40 pM (HaloTagged TFs) JF549 (Lavis Lab) for 30 min at 37°C in a 5% CO_2_ incubator and washed 3 times with ESC media without phenol-red for 5 min each. Cells were imaged in ESC media without phenol-red. Imaging was conducted on a custom-built 3i (Intelligent Imaging Innovations) microscope equipped with a Alpha Plan-Apochromat 100×/1.46 NA oil-immersion TIRF M27 objective, EM-CCD camera (Andor iXon Ultra 897), a Zeiss Definite Focus 2 system and a motorized mirror to achieve HiLo-illumination. The customized laser launch includes 405 nm (350 mW), 488 nm (300 mW), 561 nm (1 W) and 640 nm (1 W) lasers. A multi-band dichroic (405 nm/488 nm/561 nm/633 nm quad-band bandpass filter) was used to reflect a 561 nm laser into the objective and emission light was filtered using a bandpass emission filter. The laser intensity was controlled using an acousto-optic transmission filter. A low constant laser intensity was used to minimize photobleaching. Images were collected at a frame rate of 5 Hz for a total of 1000 frames. Each Halo-tagged line was imaged in three biological replicates of 5–12 cells. Examples of raw SPT videos for H2B-Halo, Halo-HSF2, and Halo-H2DBD in interphase and mitosis are shown in the Supplemental Data (Supplementary Videos 1–6).

HaloTagged TF particles were identified using SLIMfast (35), a custom-written MATLAB implementation of the MTT algorithm (36), using the following algorithm settings: localization error: 10^−6.25^; exposure time: 200 ms; deflation loops: 3; number of gaps allowed: 1; maximum number of competitors: 5; maximal expected diffusion constant (μm^2^/s): 0.5. The residence times of HaloTagged TFs were determined using custom scripts as previously described (10,15). Briefly, we quantified the dwell time of each HaloTagged TF molecule and generated a survival curve for bound HaloTagged TFs as a function of time. A two-exponential function was fitted to the survival curve to determine apparent *k*_off_ rates of HaloTagged TFs and was used to calculate the time to 1% bound shown in Figures 3B, C, 6C, and D. Photobleaching correction was performed by subtracting the apparent *k*_off_ of H2B-Halo from the apparent *k*_off_ of the HaloTagged TFs, and residence time was determined by taking the inverse of the photobleach-corrected *k*_off_ for each HaloTagged TF.

### CUT&tag

CUT&Tag assay was performed as previously described (32). Briefly, 100 000 mESCs were used per sample and were bound to Concanavalin A-coated beads (Cedarlane Labs BP531-3ML). Cryopreserved *Drosophila melanogaster* S2 cells were spiked in at a 10% concentration (10 000 S2 per 100 000 mESCs) or 20% concentration (20 000 S2 per 100 000 mESCs) for library preparation and sequencing normalization. Cells attached to beads were incubated at 4°C overnight with primary antibodies. Epicypher pAG-Tn5 (EpiCypher EP151117) was used at 1:20 final concentration and tagmentation was performed for 1 h in a 37°C water bath. DNA fragments were solubilized in STOP solution by overnight incubation in a 37°C water bath. Library preparation was also performed as in the original protocol.

The following primary antibodies were used for CUT&Tag at 1:100 dilution: α-HSF1 (Abcam, ab2923), α-HSF2 (33), α-H3K27me3 (Cell Signaling Technologies C36B11), α-SOX2 (Cedarlane 39844), α-HA tag (Epicypher, EP132010), Rabbit α-Mouse IgG (Abcam ab46540). For the secondary antibody, Guinea Pig α-Rabbit IgG (Antibodies-Online ABIN101961) at 1:100 dilution was used.

### CUT&tag analysis

Reads were mapped on mm10 genome build using Bowtie2 version 2.4.2 (37) with the following parameters: –local –very-sensitive-local –no-unal –no-mixed –no-discordant –phred33 -I 10 -X 2000. PCR duplicate reads were kept as these sites may represent real sites from adapter insertion from Tn5 as described previously (38). To quantify reads from S2 spiked in cells, reads were mapped on dm6 genome build using Bowtie2 as above with additional parameters: –no-overlap –no-dovetail. No additional filtering of mapped reads was performed. The number of reads aligned to the *D. melanogaster* genome per sample was used to calculate scaling values, where an arbitrary constant (100 000; 200 000 for 20% spike-in) was divided by the number of fly reads and normalized such that the asynchronous sample was set to 1. After alignment, BAM files were converted to bigwig files scaled with the calculated scaling values (Supplementary Table S3) using deepTools and visualized as gene tracks using IGV (39). Heatmap analyses were performed using DeepTools and BedTools suite (40,41). ComputeMatrix from deepTools was done using bin size 10. Peak calling for HSF1 and HSF2 CUT&Tag was performed using the SECAR suite (42) with options: ‘non’ and ‘stringent’, using IgG signal as the control. Bedgraph files for SECAR were prepared with the code provided with the SECAR suite. The peaks identified in asynchronous and mitotic samples were combined and filtered for unique sites to generate a BED file of binding sites. BED file of SOX2 binding sites was obtained from previous literature (43). Read counts for scatter plots were obtained for each TF from BAM files using the bedtools multicov command against a BED file with binding sites for each of TFs. Read counts were then normalized to the *D. melanogaster* scaling values and plotted using the ggplot2 suite in R. Pearson correlation analysis was performed using deepTools multBamSummary with the BED option. The Pearson correlation coefficients were plotted as a heatmap using the plotCorrelation command with the bins (default 10 kb), remove outliers, and skip zeros options.

### Putative binding sites analysis

Primary binding motifs for mouse HSF1, HSF2, and SOX2 were obtained from HOCOMOCO database (44) as Position Weight Matrices (PWMs). PWMs were then used as an input motif in the FIMO tool (45) to identify putative binding sites in the mouse (mm10) genome with p-value threshold set below 1 × 10^−4^.

### Statistical analyses

Unless stated otherwise, *P*-values were calculated using a standard two-tailed t-test with a 95% confidence interval using GraphPad Prism suite. For all chromatin enrichment data, each biological replicate was averaged, and *P*-values were calculated between these averages for each sample (not between the technical replicates that are shown on the chromatin enrichment graphs).

## RESULTS

### HSF1 and HSF2 display different degrees of site-specific DNA interaction during mitosis

To investigate the binding behaviors of endogenous HSF1 and HSF2 in mESCs during mitosis without crosslinking, we performed spike-in normalized CUT&Tag, an assay that maps protein binding to DNA in native conditions (32). We collected synchronized mitotic cells by treating mESCs with nocodazole, a tubulin polymerization inhibitor (46), for 6 h followed by shake-off. We assessed the purity of mitotic cells using flow cytometry, which shows that the mitotic index (percentage of mitotic cells) was above 91% (Supplementary Figure S1B, C).

**Figure 1.**
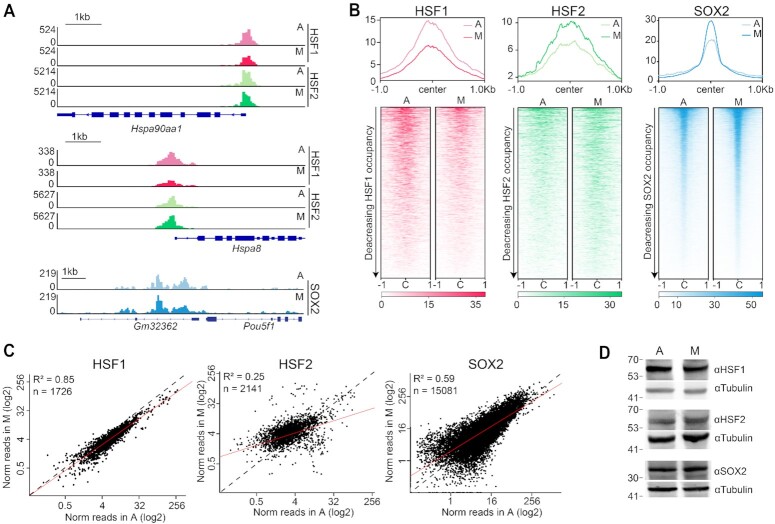
HSF1 and HSF2 have different degrees of DNA-binding activity in mitosis. (**A**) Gene browser tracks of HSF1 and HSF2 over *Hsp90aa1* and *Hspa8* promoters, and SOX2 over *Pou5f1* (OCT4) promoter in asynchronous (A) and mitotic (M) cells. (**B**) Genome-wide average plots (top) and heatmaps (bottom) of HSF1 (left), HSF2 (middle), and SOX2 (right) CUT&Tag in asynchronous (A) and mitotic (M) cells. CUT&Tag signal was calculated in a 2 kb window surrounding binding sites of the respective TF. For heatmaps, binding sites were ordered by decreasing occupancy of the given TF. (**C**) Normalized read counts of HSF1 (left), HSF2 (middle), and SOX2 (right) for each binding site in asynchronous (A) and mitotic (M) cells are displayed as a scatter plot. N denotes number of analyzed sites. (**D**) HSF1 (top), HSF2 (middle) and SOX2 (bottom) protein levels in asynchronous (A) and mitotic cells (M). Tubulin is shown as a loading control.

First, we examined SOX2, a transcription factor with established mitotic DNA-binding activity (10,12,14,47), although with some discrepancies between studies. CUT&Tag analysis shows high enrichment of SOX2 at known binding sites, such as at the proximal enhancer of the *Pou5f1* gene in asynchronous samples, and this high level of binding is maintained in mitotic cells (Figure 1A) with high reproducibility between replicates (Supplementary Figure S2A–C). To assess mitotic SOX2 binding genome-wide, we plotted the CUT&Tag signal for previously identified SOX2 binding sites (43) as heatmaps (Figure 1B) and displayed the normalized read counts per binding site for asynchronous and mitotic cells as a scatter plot (Figure 1C). Both analyses show that SOX2 maintains a high level of site-specific binding in mitosis genome-wide, consistent with previously published studies (14). Therefore, CUT&Tag can measure endogenous TF binding during mitosis.

Next, we performed CUT&Tag on HSF1 and HSF2 in mitotic and asynchronous cells. We observed strong binding profiles at known HSF1 and HSF2 binding sites in asynchronous cells, including *Hsp90aa1* and *Hspa8* (Figure 1A), with strong concordance between replicates (Supplementary Figure S2A–C). In mitotic samples, we observed decreased HSF1 binding at specific loci, including *Hspa8*, though, unexpectedly, many sites remained bound, such as *Hsp90aa1*. Consistent with previous studies, HSF2 binding at specific loci, such as *Hsp90aa1* and *Hspa8*, was generally maintained, or even increased (7,9,25). Using SEACR, a CUT&Tag-specific peak calling algorithm, we identified combined binding sites in asynchronous and mitotic samples for HSF1 (1726) and HSF2 (2141). HSF2 binds a smaller number of genomic loci than some bookmarking factors, such as SOX2 (15081, Figure 1C) and ESRRB (14559) (17), but a similar number as others, like FOXA1 (3596) (16). We then plotted HSF1and HSF2 CUT&Tag signal for asynchronous and mitotic samples in a 2-kb region surrounding these identified binding sites as heatmaps and average profiles (Figure 1B) and displayed the normalized read counts per binding site as a scatter plot (Figure 1C). Globally, the average signal of HSF1 binding in mitosis was lower than in asynchronous cells, but binding was maintained to some degree (Figure 1B and C). In contrast, HSF2 CUT&Tag signal was maintained or even increased genome-wide during mitosis (Figure 1B and C). The differing degrees of mitotic binding between HSF1 and HSF2 is not due to altered protein levels as HSF1, HSF2 and SOX2 show no change in expression during mitosis (Figure 1D). Our results for HSF1 contrast with previously published data (25), though the differences could be due to cell type or the use of cross-linking based ChIP assays.

Since the mitotic synchronization contained a low level of non-mitotic cell contamination, we examined the contribution of such contamination to the CUT&Tag data. We generated *Hsf2* knockout (KO) mESCs (*Hsf2^−/−^*; Supplementary Figure S3A and B) and performed HSF2 CUT&Tag on varying mixed ratios of wild type (WT) and *Hsf2*^−/−^ mESCs (Supplementary Figure S3E–G). These analyses show that even with 10% of WT cells mixed with *Hsf2*^−/−^ mESCs, the HSF2 CUT&Tag signal remains very low, suggesting that even 10% contamination does not significantly contribute to the CUT&Tag signal. Taken together, these results confirm that CUT&Tag can measure site-specific binding in mitotic cells, and that HSF1 and HSF2 have varying degrees of mitotic binding in mESCs.

### HaloTagged HSFs are excluded from mitotic chromosomes when examined with live-cell imaging

**Figure 2.**
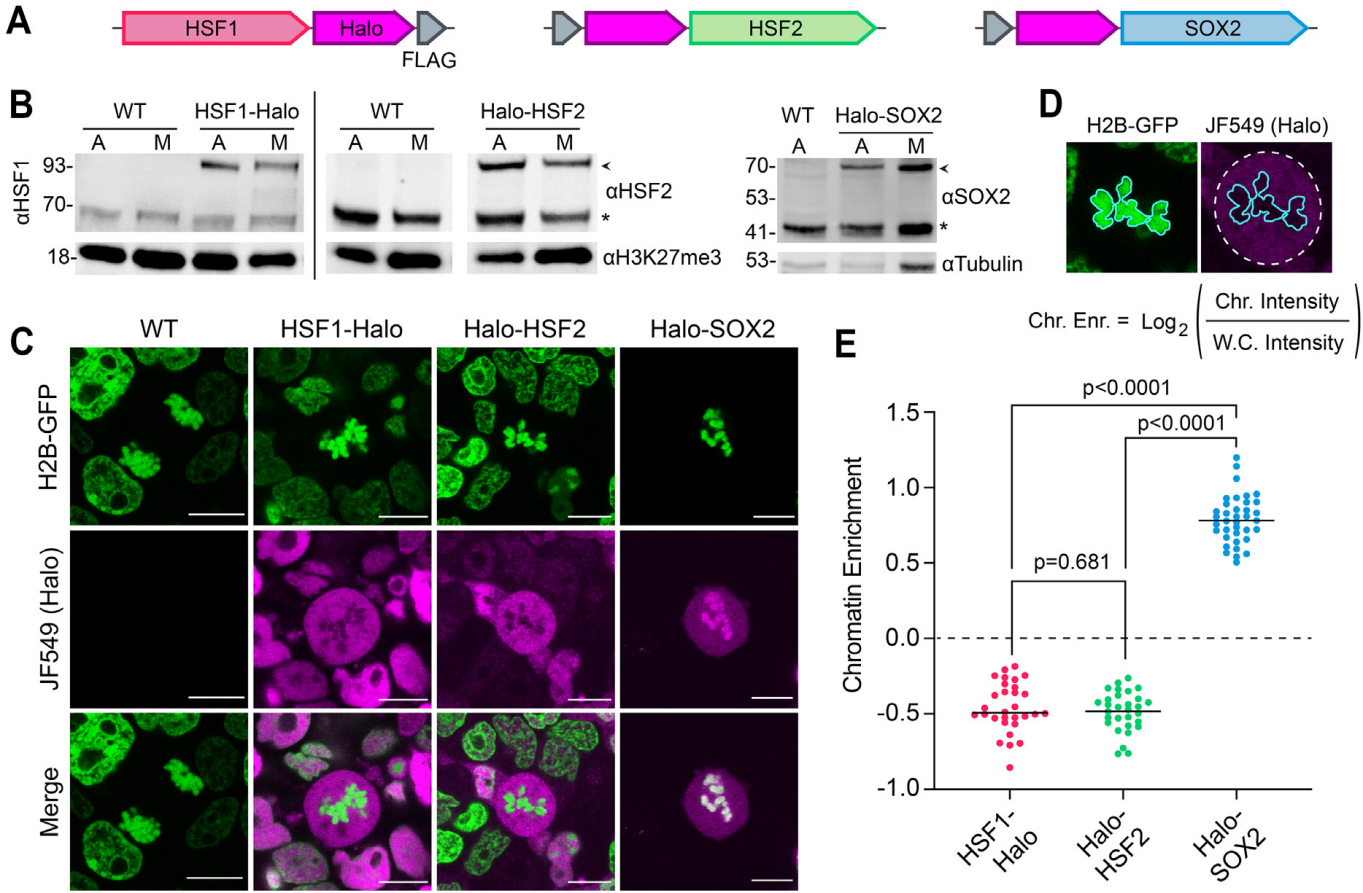
HaloTagged HSFs are excluded from mitotic chromosomes when viewed with live-cell imaging. (**A**) Schematics of stably integrated HaloTag-TF overexpression constructs in WT JM8 cells expressing H2B-GFP. Each TF (SOX2 in blue, HSF1 in red, and HSF2 in green) is tagged with HaloTag (magenta) and FLAG Tag (gray) and expressed under an EF1α promoter. (**B**) Western blots of each HaloTag-TF construct in asynchronous (A) and mitotic (M) cells using α-HSF1 (left), α-HSF2 (middle) and α-SOX2 (right). Mitotic cell populations were synchronized with 50 ng/ml nocodazole for 6 h before collecting cells via shake-off. Asterisks indicate endogenous TFs (HSF1, HSF2, SOX2). Arrows indicate HaloTagged TFs (HSF1-Halo, Halo-HSF2, and Halo-SOX2). H3K27me3 and Tubulin are shown as loading controls. (**C**) Live-cell fluorescent imaging of HaloTag-TF constructs (magenta) labeled with 200 nM JF549 dye. DNA is visualized with H2B-GFP overexpression (green). Scale bars represent 10 μm. (**D**) Strategy for quantifying TF chromatin enrichment. (**E**) Chromatin enrichment quantification for the indicated HaloTagged TFs (*n* = 30 cells for HSF1-Halo, 30 for Halo-HSF2, and 37 for Halo-SOX2 across three biological replicates). Data are visualized as individual data points with mean value indicated.

We next explored how HSF1 and HSF2 interact with mitotic chromatin using live-cell imaging. We stably integrated HaloTagged constructs of each TF expressed under the EF1α promoter (HSF1-Halo, Halo-HSF2, Halo-SOX2; Figure 2A) into WT JM8 mESCs stably expressing Histone 2B tagged with GFP (H2B-GFP) and verified the expression by Western blot analysis (Figure 2B). C-terminal tagging of HSF2 resulted in degradation of the protein, thus it was tagged N-terminally (Supplementary Figure S4A). A cell line stably overexpressing HaloTagged SOX2 was used as control for coating behavior. HaloTagged TF overexpression cell lines were stained with Halo-specific JF549 dye (48), and imaged under live-cell conditions. Chromatin enrichment was quantified by calculating the log_2_ ratio of the mean HaloTag signal intensity on mitotic chromosomes over the signal intensity across the whole cell (Figure 2D). As previously observed, Halo-SOX2 is strongly enriched on mitotic DNA (Figure 2C and E). Given the reduced specific mitotic binding of endogenous HSF1, we expected and observed that HSF1-Halo is excluded from mitotic DNA (Figure 2C and E). Surprisingly, Halo-HSF2 is excluded to a similar degree as HSF1-Halo (Figure 2C and E). We next examined if the reduced coating is due to differences in putative binding sites. A computational prediction of the number of potential binding sites genome-wide using consensus binding motifs revealed roughly similar numbers of putative sites between HSF1 and HSF2 (61 078 and 74 017, respectively), which are also similar to SOX2 (60812) (Supplementary Figure S4B). Therefore, the difference in coating behavior between SOX2 and the HSFs is not attributable to decreased putative binding sites.

To exclude the possibility that competition between the endogenous and Halo-tagged HSFs is occurring, we generated *Hsf1*^−/−^ mESCs (Supplementary Figure S3C and D) in addition to the *Hsf2*^−/−^ mESCs (Supplementary Figure S3A and B). We then stably over-expressed Halo-HSF2 and HSF1-Halo in the corresponding KO cells and performed live-cell imaging (Supplementary Figure S4C). Chromatin enrichment values are highly similar to those in WT JM8 mESCs (Supplementary Figure S4D). Therefore, exclusion of HSF1-Halo and Halo-HSF2 is not due to competition with endogenous HSF proteins. Exclusion of the HSFs is likely not due to the addition of the HaloTag, which was used to examine the mitotic enrichment of many other TFs in a previous study, including SOX2, SP1, OCT4 and ESRRB (10). In this same study, tagging the aforementioned TFs with an alternative fluorescent tag (mCherry) did not change the chromatin enrichment results. Therefore, unlike Halo-SOX2, Halo-HSF2 and HSF1-Halo both appear excluded from mitotic chromosomes despite having various degrees of specific mitotic binding by the endogenous HSFs as measured by CUT&Tag (Figure 1). These results confirm that site-specific mitotic binding and coating are independent properties that are measurable by different assays (12) and that, at least for the HSFs, are driven by distinct mechanisms.

### HaloTagged HSFs are more dynamic in mitosis than in interphase

**Figure 3.**
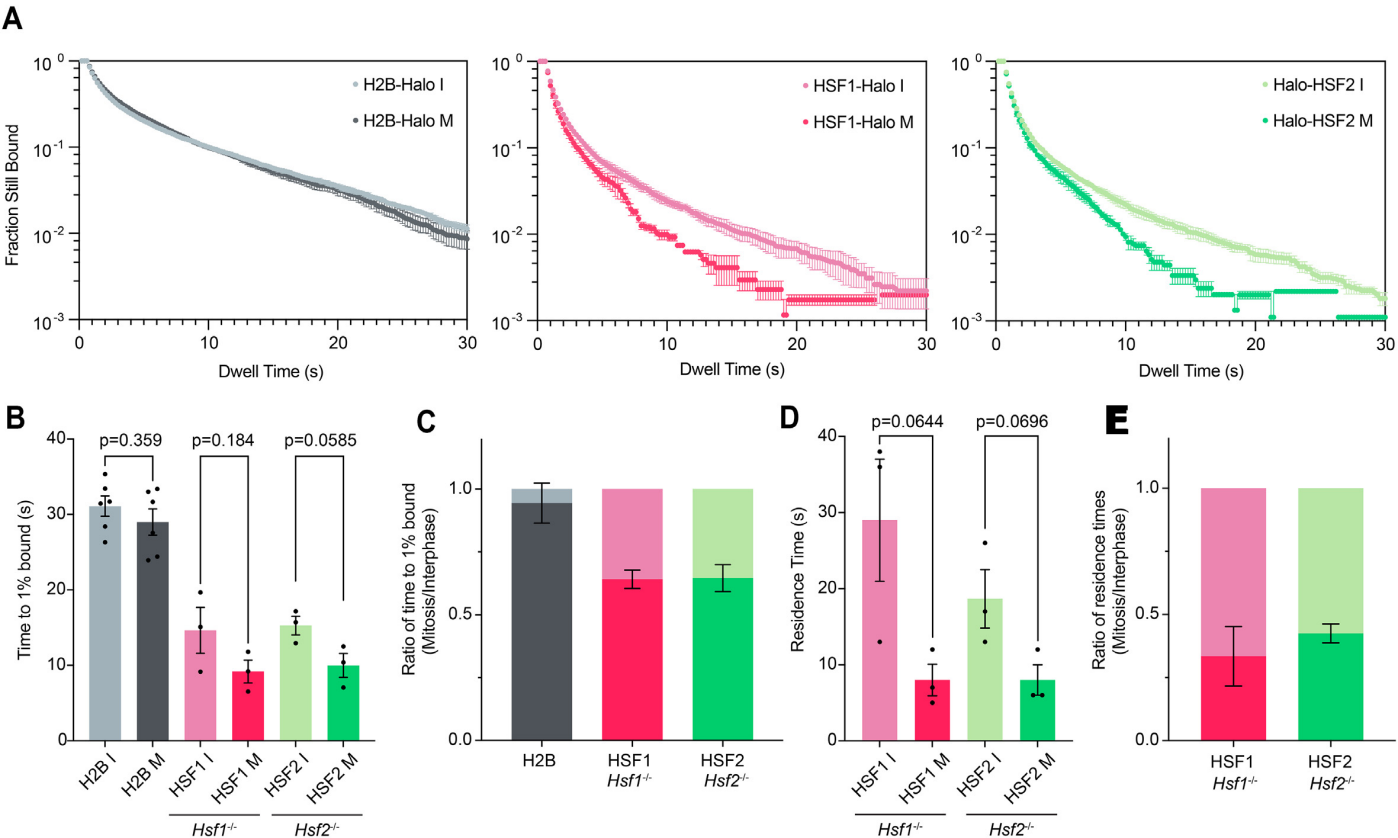
Single-particle tracking reveals that HaloTagged HSF constructs are more dynamic in mitosis than in interphase. (**A**) Dwell time curves of H2B-Halo control (gray, left), HSF1-Halo in *Hsf1*^−/−^ (red, middle), and Halo-HSF2 in *Hsf2*^−/−^(green, right) showing the fraction of HaloTagged molecules bound to DNA in interphase (I, lighter color) and mitosis (M, darker color) over time. Data depicted as mean ± SEM (*n* = 62 cells for H2B-Halo I, 60 for H2B-Halo M, 34 for HSF1-Halo I, 23 for HSF1-Halo M, 32 for Halo-HSF2 I, and 29 for Halo-HSF2 M across three biological replicates). (**B**) Time to 1% of total HaloTagged molecules bound to DNA for each cell line in (A) in interphase and mitosis, where each dot represents one biological replicate. Data depicted as mean ± SEM. (**C**) Ratio of the time to 1% of total HaloTagged molecules bound during mitosis relative to interphase. Data depicted as mean ± SEM. (**D**) Residence times of HSF1-Halo and Halo-HSF2 on DNA in interphase and mitosis, where each dot represents one biological replicate. Data depicted as mean ± SEM. (**E**) Ratio of mitotic residence times for HSF1-Halo and Halo-HSF2 relative to interphase. Data depicted as mean ± SEM.

An alternative method for quantifying DNA-TF interactions is single-molecule live-cell imaging coupled with single particle tracking (SPT) (49,50). Using sparse labeling combined with long exposure times (200 ms; slow-tracking mode), diffusing molecules ‘blur’ out while DNA-interacting molecules appear as diffraction-limited spots (10,49). Using this method, previous studies have shown that the interaction of Halo-SOX2 with mitotic DNA is more dynamic than with interphase chromatin, despite its ability to coat. To characterize the interaction dynamics of the HSFs with mitotic DNA, we performed SPT on HSF1-Halo in *Hsf1*^­/−^ mESCs, and on Halo-HSF2 in *Hsf2*^−/−^ mESCs in interphase and mitotic cells. The KO background ensures that the dynamics of the tagged HSFs are independent of the trimerization with untagged endogenous HSFs (51). After image collection, stable molecules are localized and tracked using the SLIMfast algorithm (35). The results are plotted as a log histogram of dwell times as previously described (10,49) and averaged across three biological replicates (Figure 3A), with high consistency between replicates (Supplementary Figure S5A and B). Cells stably expressing H2B-Halo are used as a photobleaching control as previously described (10) and analyzed similarly. A two-component exponential decay model is fitted to the dwell time curves, from which the time to reach 1% of molecules still bound was calculated (Figure 3B and C). Both HaloTagged HSFs are more dynamic in mitosis than in interphase, and the dynamics between the two are nearly indistinguishable (Figure 3A and B).

To estimate an average residence time for the HaloTagged HSFs, the apparent *k*_off_ for each factor was extracted from the two-component exponential decay model as previously reported (10,49) and corrected for photobleaching using H2B-Halo (Supplementary Figure S5C). The residence time was calculated by taking the inverse of this corrected *k*_off_ (Figure 3D). Though their overall dynamics are similar, a moderate difference in the residence time in mitosis relative to interphase is observed between HSF1-Halo and Halo-HSF2 (33.4% and 42.5%, respectively, Figure 3E), though both relative residence times are less than that of SOX2 (54%) (10). Therefore, consistent with previous studies on SOX2 and other TFs showing increased dynamics during mitosis, both HSF1-Halo and Halo-HSF2 are more dynamic in mitosis than interphase despite the difference in specific binding levels (Figure 1). These data suggest that increased TF dynamics may be inherent to the mitotic cell cycle phase and independent of site-specific binding or coating properties.

### Nuclear import and export activity is not sufficient to induce HSF2 coating on mitotic chromosomes

**Figure 4.**
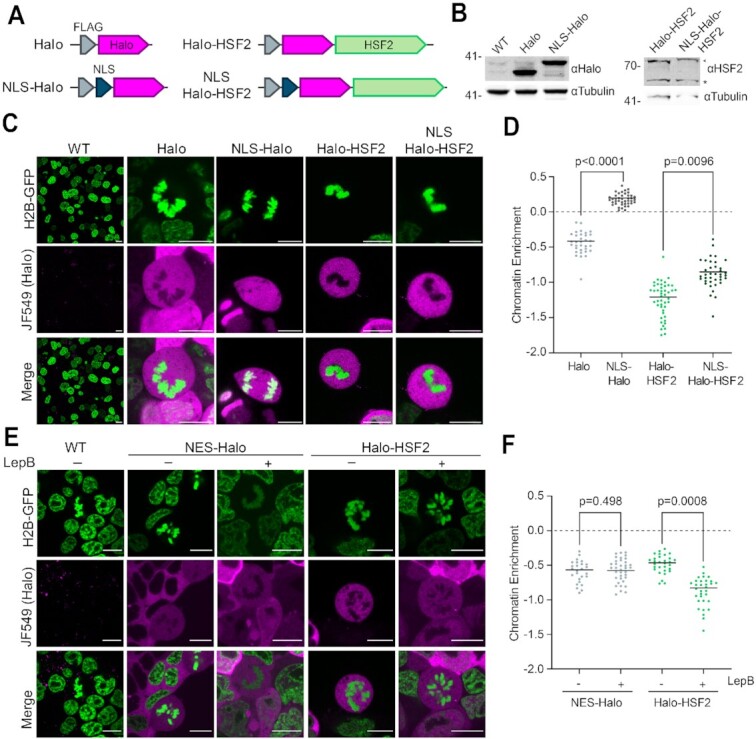
Nuclear import and export activity is not sufficient to induce HSF2 coating on mitotic chromosomes. (**A**) Schematics of stably integrated HaloTag overexpression constructs in WT JM8 cells expressing H2B-GFP, as in Figure 2A. (**B**) Protein levels of Halo and NLS-Halo constructs using α-Halo (left), and Halo-HSF2 and NLS-Halo-HSF2 constructs using α-HSF2 (right). Asterisk indicates endogenous HSF2, and arrow indicates HaloTagged protein. Tubulin is shown as a loading control. (**C, E**) Live-cell imaging of cells labeled with 200 nM JF549 dye (magenta) expressing indicated HaloTagged constructs as well as control cells (WT). DNA is visualized by H2B-GFP overexpression (green). Scale bars are 10μm. For (**E**) cells, were either treated with 10 ng/ml Leptomycin B (LepB) (+) or with vehicle (–) for 1h before imaging. (**D, F**) Chromatin enrichment quantification for the indicated Halo constructs (*n* = 33 cells for Halo, 42 for NLS-Halo, 46 for Halo-HSF2, 38 for NLS-Halo-HSF2, 26 for NES-Halo control, 36 for NES-Halo + LepB, 30 for Halo-HSF2 control, and 32 for Halo-HSF2 + LepB across 3 biological replicates). Data are visualized as individual data points with mean value indicated.

We next questioned what prevents Halo-HSF2 from coating mitotic chromosomes. Previous studies have shown that, despite disassembly of the nuclear envelope during mitosis, the nuclear localization signal (NLS) and the nuclear import mechanism play a role in localizing proteins to mitotic chromosomes (10,26,52). To test the possibility that the NLS within HSF2 is insufficient to enforce coating of mitotic DNA, we added three repeats of the SV40 NLS to Halo-HSF2 and to HaloTag as a control (Figure 4A) and confirmed overexpression of each construct in WT JM8 mESCs (Figure 4B). Using live-cell imaging, we quantified the enrichment on mitotic chromosomes. Addition of the SV40 NLS to HaloTag induced coating on mitotic DNA (Figure 4C and D), in accordance with previous reports (10). However, addition of the SV40 NLS to Halo-HSF2 did not cause it to localize to mitotic DNA (Figure 4C and D). This data suggests that, unlike for HaloTag, the SV40 NLS is insufficient to alter Halo-HSF2 coating behavior during mitosis.

Protein localization into the nucleus is the result of two opposing mechanisms–nuclear import and export (53,54). We next examined the role of nuclear export in this process using the export inhibitor Leptomycin B (LepB) (55,56). To validate LepB activity, we expressed HaloTag fused to the HIV-1 Rev Nuclear Export Signal (NES). This construct is cytoplasmic in untreated interphase cells, and becomes visible in the nucleus after LepB treatment, confirming drug activity (Supplementary Figure S6A and B). We labeled mESCs expressing Halo-HSF2 with JF549 and treated the cells for 1h with LepB prior to live-cell imaging. Although the mean chromatin enrichment value for Halo-HSF2 in cells treated with LepB was much lower than those in untreated cells (Figure 4F), overall LepB treatment had no effect on Halo-HSF2 exclusion from mitotic DNA (Figure 4E and F). These results are consistent with the addition of the SV40 NLS described above, suggesting that neither the nuclear import or export mechanisms determine the eviction of Halo-HSF2 from chromatin during cell division.

### The heptad repeats A and B of the HSFs are necessary for exclusion from mitotic chromosomes

**Figure 5.**
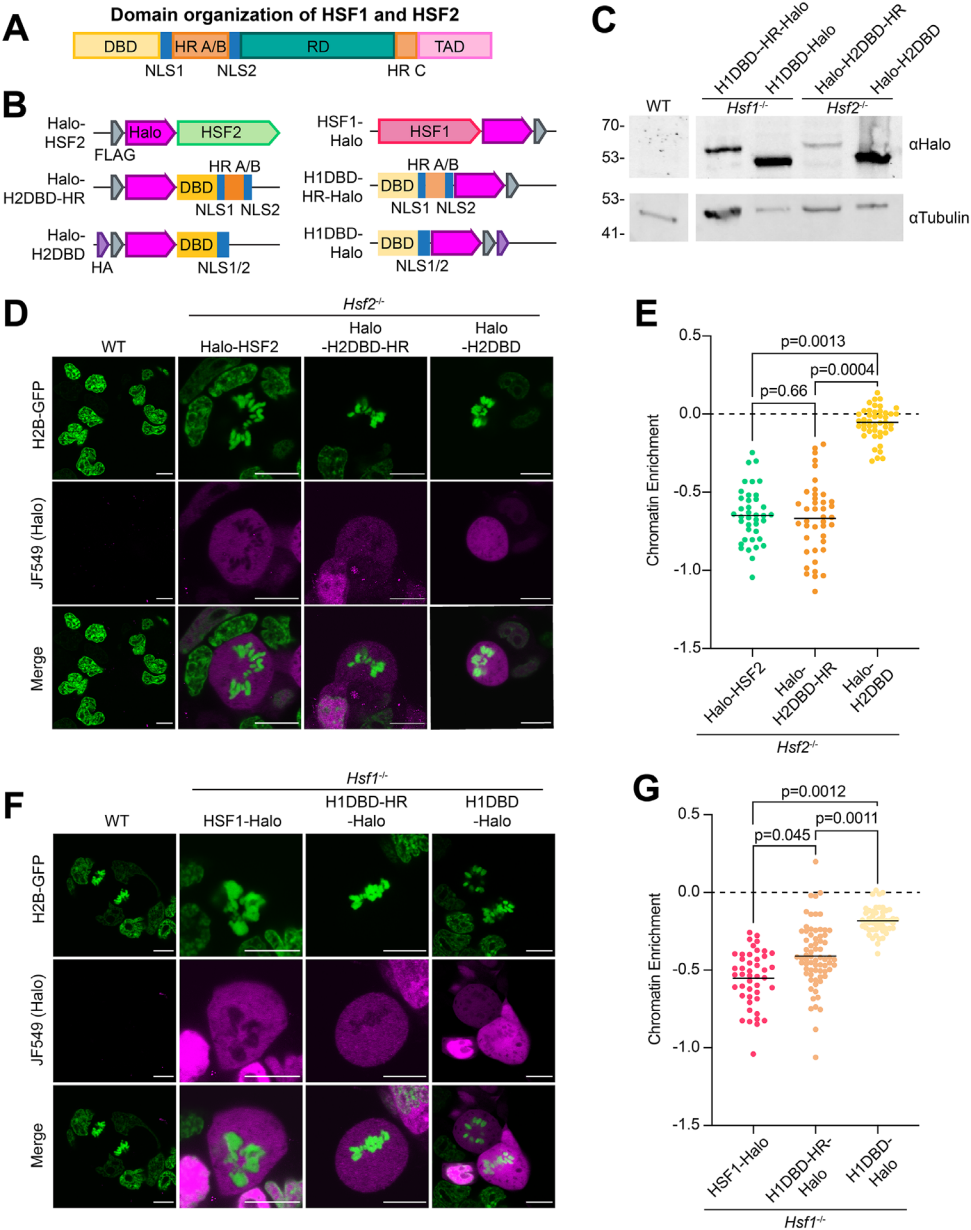
The HR-A/B region of HSF1 and HSF2 is necessary for exclusion from mitotic chromosomes. (**A**) Schematic showing the analogous functional domain organization of HSF1 and HSF2. Domains include the DBD (yellow), two NLS sequences (blue), HR regions A, B and C (orange), the regulatory domain (teal), and the transactivation domain (pink). (**B**) Schematics of stably integrated HaloTag overexpression constructs in either *Hsf2*^−/−^(Halo-HSF2 and truncations) or *Hsf1*^−/−^ (HSF1-Halo and truncations) cells expressing H2B-GFP, as in Figure 2A. (**C**) Western blots of HaloTagged truncation constructs using α-Halo. Tubulin is shown as a loading control. (**D**) Live-cell fluorescent imaging of HaloTagged constructs (magenta) labeled with 200 nM JF549 dye. DNA is visualized with H2B-GFP overexpression (green). Scale bars represent 10 μm. (**E**) Chromatin enrichment quantification for the indicated HaloTagged constructs (*n* = 39 cells for Halo-HSF2, 41 for Halo-H2DBD-HR, and 45 for Halo-H2DBD across three biological replicates). (**F**) Live-cell fluorescent imaging as in (C). (**G**) Chromatin enrichment quantification for the indicated HaloTagged constructs (*n* = 45 cells for HSF1-Halo, 70 for H1DBD-HR-Halo and 51 for H1DBD-Halo across three biological replicates).

Next, we examined if the inability of Halo-HSF2 to coat mitotic DNA may be an intrinsic property. The HSFs contain multiple functional domains (Figure 5A): the DBD, two nuclear localization signals (NLS1 and NLS2), three heptad repeat (HR) multimerization regions (A, B and C), a regulatory domain (RD), and a transactivation domain (TAD) (29). Apart from the DBD, the HR regions are the most relevant to DNA binding since they contribute to the trimerization of the protein, which is necessary for site-specific binding (57). HR-A/B is essential for trimerization, while HR-C inhibits trimerization (58).

To identify regions that may inhibit coating, we made two truncations of Halo-HSF2 (Figure 5B). First, we removed the TAD, RD and HR-C of HSF2 to generate the Halo-HSF2DBD-HRA/B-NLS construct (Halo-H2DBD-HR), which contains the DBD, NLSs and the HR-A/B domains. Second, this construct was further truncated by removing HR-A/B to generate the Halo-HSF2DBD-NLS construct (Halo-H2DBD). Each truncation was stably overexpressed in *Hsf2^−/−^* mESCs expressing H2B-GFP and verified with western blots against HaloTag (Figure 5C). The truncation constructs were imaged in live cells alongside the full-length Halo-HSF2. Removing the TAD, RD, and HR-C (Halo-H2DBD-HR) did not increase chromatin enrichment (Figure 5D and E). However, once HR-A/B was removed, we observed a dramatic increase in chromatin coating (Figure 5D and E), though the construct is still less enriched than Halo-SOX2 (Figure 2C and E). These results demonstrate that the HaloTag is not causing eviction of the protein, as the Halo-H2DBD gains the capacity to coat chromosomes. The behavior of the truncated proteins is not affected by the presence of endogenous HSF2; live-cell imaging of Halo-H2DBD-HR and Halo-H2DBD in WT JM8 cells show chromatin enrichment values highly similar to those in *Hsf2*^−/−^ mESCs (Supplementary Figure S6C and D). More importantly, we show that the HR-A/B domain is necessary for the exclusion of Halo-HSF2 from mitotic DNA, though its effect on site-specific binding remains to be seen.

To parallel the truncation of HSF2, we generated two analogous HSF1 truncation constructs (H1DBD-Halo and H1DBD-HR-Halo, Figure 5B). Both H1DBD-Halo and H1DBD-HR-Halo were overexpressed in *Hsf1*^−/−^ cells (Figure 5C) and imaged live (Figure 5F). These truncations follow a similar pattern as the HSF2 truncations: the chromatin enrichment of H1DBD-Halo is much greater than H1DBD-HR-Halo (Figure 5G). Interestingly, the chromatin enrichment of H1DBD-HR-Halo is increased in comparison to the full length HSF1-Halo (Figure 5G), an effect not observed with the HSF2 truncations (Figure 5E). However, H1DBD-HR-Halo was still visibly excluded, and its enrichment did not increase to the same extent as the DBD alone. Therefore, the non-DBD domains of HSF1, particularly the HR-A/B domain, dictate its exclusion from mitotic chromatin, similar to what is seen with HSF2.

### The role of the DBD in dynamics and DNA binding

**Figure 6.**
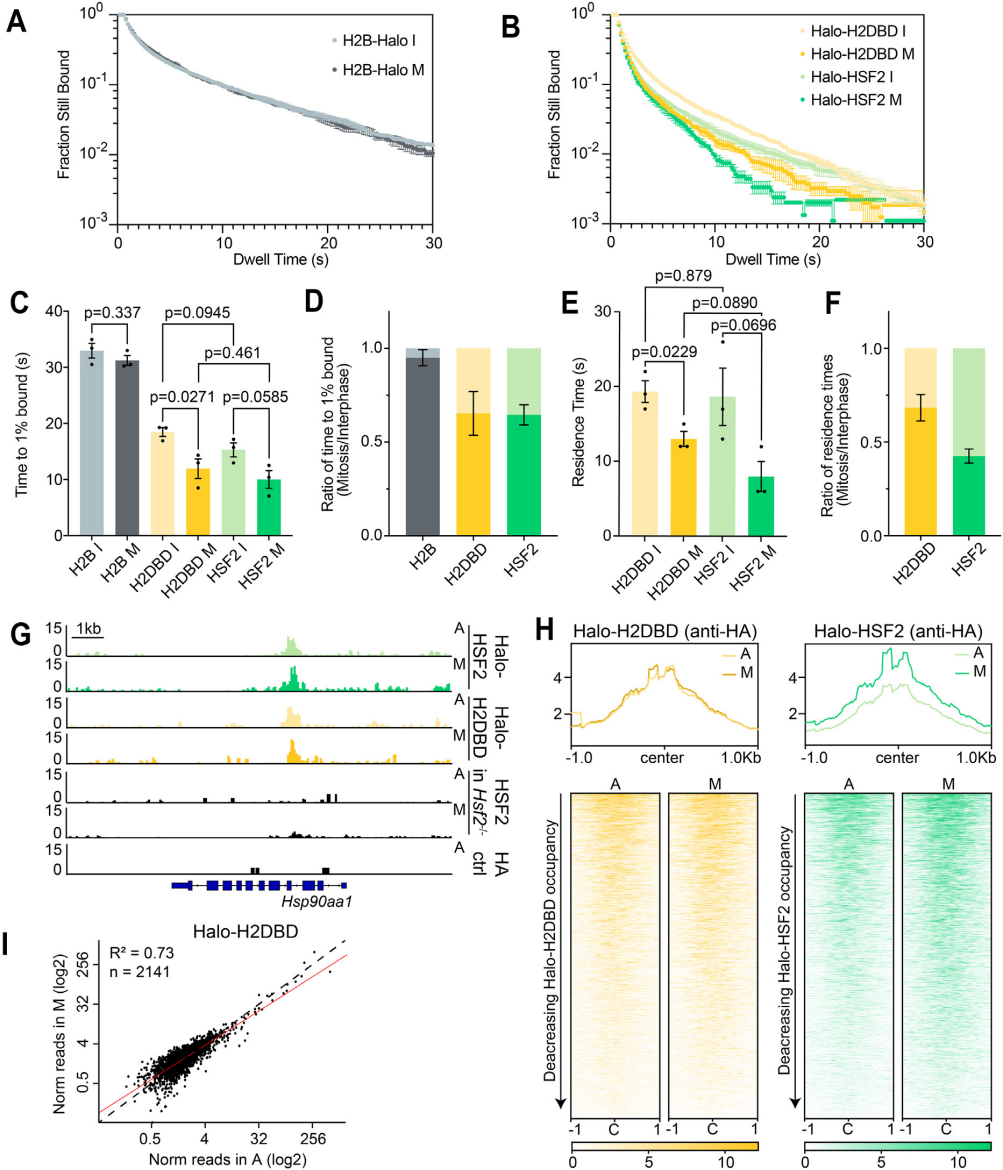
Role of the DBD in dynamics and DNA binding. (**A**) Decay curves of H2B-Halo control showing the fraction of HaloTagged molecules bound to DNA in interphase (I, light gray) and mitosis (M, dark gray) over time. Data depicted as mean ± SEM (*n* = 31 cells for H2B-Halo I and 31 for H2B-Halo M across three biological replicates). (**B**) Decay curves of Halo-H2DBD (yellow), and Halo-HSF2 (green, data as seen in Figure 3A) showing the fraction of HaloTagged molecules bound to DNA in interphase (I, lighter color) and mitosis (M, darker color) over time. Data depicted as mean ± SEM (*n* = 34 for Halo-H2DBD I, and 29 for Halo-H2DBD M across three biological replicates). (**C**) Time to 1% of total HaloTagged molecules bound to DNA for H2B-Halo control, Halo-H2DBD, and Halo-HSF2 (data as in Figure 3B) in interphase and mitosis, where each dot represents one biological replicate. Data depicted as mean ± SEM. (**D**) Ratio of the time to 1% of total HaloTagged molecules bound during mitosis relative to interphase. Data depicted as mean ± SEM. (**E**) Residence times of Halo-H2DBD and Halo-HSF2 (data as in Figure 3D) on DNA in interphase and mitosis, where each dot represents one biological replicate. Data depicted as mean ± SEM. (**F**) Ratio of mitotic residence times relative to interphase. Data depicted as mean ± SEM. (**G**) Gene browser tracks of Halo-H2DBD and HA-tagged Halo-HSF2 over *Hsp90aa1* locus in asynchronous (A) and mitotic (M) cells. (**H**) Genome-wide average plots (top) and heatmaps (bottom) of Halo-H2DBD (left), and HA-tagged Halo-HSF2 (right) CUT&Tag in asynchronous (A) and mitotic (M) cells. CUT&Tag signal was calculated in a 2 kb window surrounding HSF2 binding sites. For heatmaps, binding sites were ordered by decreasing occupancy of the given construct. (**I**) Normalized read counts of Halo-H2DBD for each binding site in asynchronous (A) and mitotic (M) cells displayed as a scatter plot. *N* denotes number of analyzed sites.

We next examined if the change in coating behavior affects mitotic DNA interaction dynamics for our strongest-coating truncation, Halo-H2DBD, by performing single-molecule live-cell imaging followed by SPT in WT JM8 cells. The dwell time curves for Halo-H2DBD reveal that the truncation is less dynamic in both interphase and mitosis than the full-length protein, with Halo-HSF2 data replotted for comparison (Figure 6B; replicate data shown in Supplementary Figure S7A). Dwell curves for the H2B-Halo control are seen in Figure 6A. The dwell curves were fitted with a two-component exponential decay model and quantified as the time to 1% of molecules bound (Figure 6C and D). The difference between the time to 1% bound in mitosis and interphase for Halo-H2DBD follows a similar trend as the full-length Halo-HSF2, where the interactions of the TF with DNA are more dynamic during mitosis compared to interphase (Figure 6C). Examining the residence times and corresponding *k*_off_ values of the two proteins, Halo-H2DBD consistently binds mitotic DNA for longer than Halo-HSF2 (mean = 13 s and 8 s, respectively), despite having similar interphase residence times (mean = 18.7 s and 19.3 s, respectively) (Figure 6E). Reciprocally, the mean *k*_off_ in mitosis is 0.0796 s^−1^ for Halo-H2DBD and 0.138 s^−1^ for Halo-HSF2, though the mean *k*_off_ values in interphase are similar (0.052 s^−1^ for Halo-H2DBD and 0.059 s^−1^ for Halo-HSF2) (Supplementary Figure S7B). This difference is also apparent in the ratio of residence times, where Halo-HSF2 mitotic binding is 42.5% relative to interphase and Halo-H2DBD mitotic binding is 68.3% relative to interphase (Figure 6F). This data suggests that the coating ability of Halo-H2DBD changes the mitotic residence time of the construct when compared to full-length Halo-HSF2, although the truncation remains more dynamic in mitosis than in interphase.

Given the change in coating behavior, we tested whether Halo-H2DBD is able to establish site-specific interactions with DNA by performing α-HA CUT&Tag on Halo-H2DBD and on the overexpressed HA-tagged Halo-HSF2 in asynchronous and mitotic *Hsf2*^−/−^ cells. Remarkably, we observed that the Halo-H2DBD binding profile is highly similar to full-length Halo-HSF2 at specific binding sites both in asynchronous and mitotic cells, including the well-known heat shock gene *Hsp90aa1* (Figure 6G). We then plotted CUT&Tag signal for asynchronous and mitotic samples in a 2-kb region surrounding HSF2 binding sites as heatmaps and average profiles (Figure 6H) and displayed the normalized read counts per binding site as a scatter plot (Figure 6I). Globally, Halo-H2DBD also maintains specific binding in mitosis to a similar degree as the full-length Halo-HSF2 (Figure 6H and I), with high similarity between replicates (Supplementary Figure S8A and B). Therefore, the non-DBD domains of HSF2 affect its ability to coat mitotic chromatin but not its specific mitotic binding ability.

### The HSF DBDs are insufficient to induce mitotic coating, in contrast to SOX TFs

**Figure 7.**
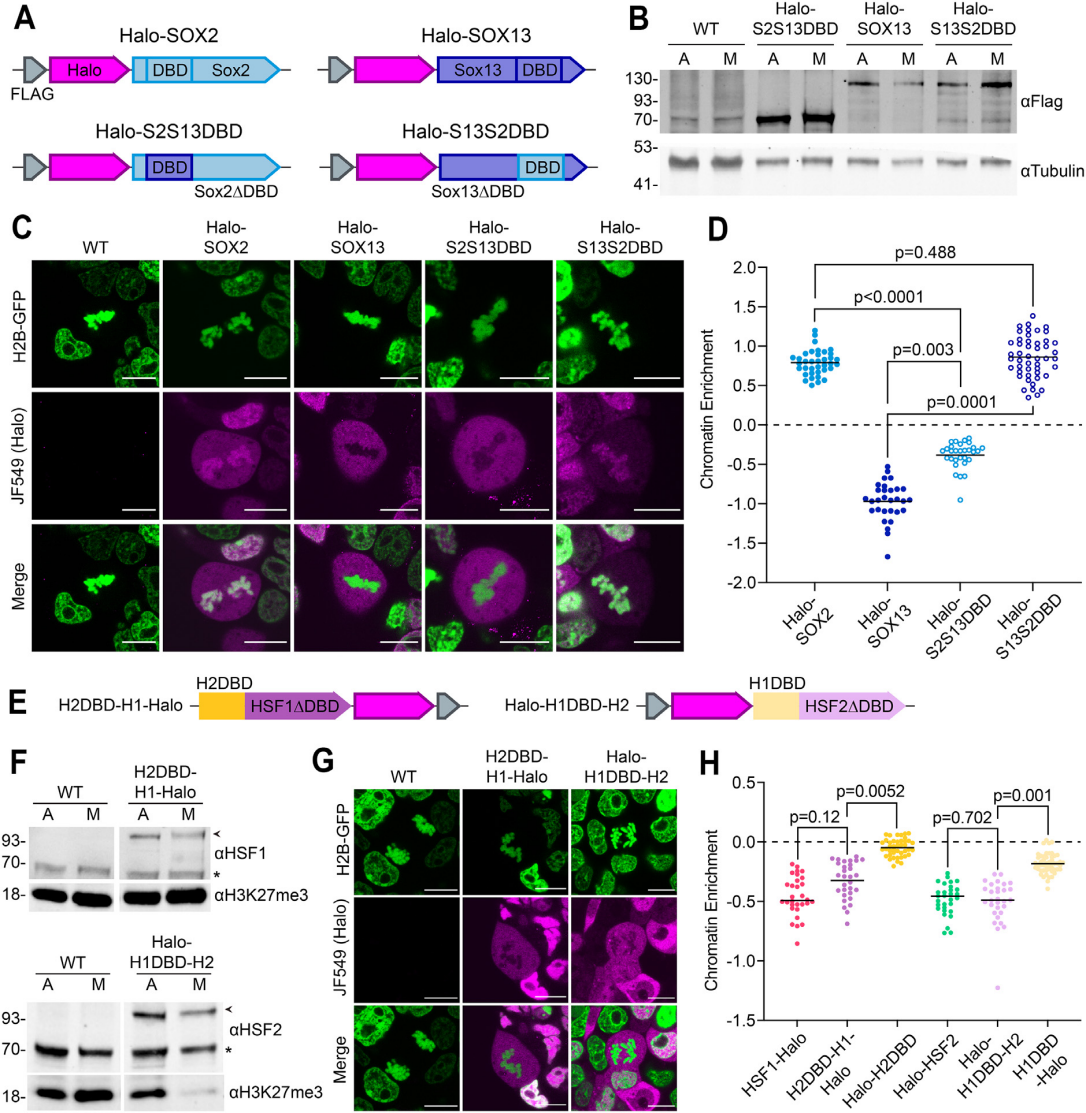
The HSF DBDs are not sufficient to induce mitotic coating, in contrast to SOX TFs. (**A**) Schematics of stably integrated HaloTag overexpression constructs in WT JM8 cells expressing H2B-GFP, as in Figure 2A. (**B**) Western blots of each HaloTag-TF construct in asynchronous (A) and mitotic (M) cells using α-Flag. Tubulin is shown as a loading control. Mitotic cell populations were synchronized with 50 ng/ml nocodazole for 6 h before collecting cells via shake-off. (**C**) Live-cell fluorescent imaging of HaloTag-TF constructs (magenta) labeled with 200 nM JF549 dye. DNA is visualized with H2B-GFP overexpression (green). Scale bars represent 10 μm. (**D**) Chromatin enrichment quantification for the indicated HaloTagged TFs (n = 37 cells for Halo-SOX2, 30 for Halo-SOX13, 30 for Halo-S2S13DBD, and 48 for S13S2DBD across three biological replicates). (**E**) Schematics of H2DBD-H1-Halo and Halo-H1DBD-H2 constructs which were stably integrated into WT JM8 cells expressing H2B-GFP. (**F**) Western blot of H2DBD-H1-Halo and Halo-H1DBD-H2 in asynchronous (A) and mitotic (M) cells using α-HSF1 and α-HSF2. Asterisks indicate endogenous HSFs. Arrows indicate HaloTagged chimeric constructs. H3K27me3 shown as a loading control. Mitotic cell populations were synchronized as in (B). (**G**) Live-cell fluorescent imaging as in (C). (**H**) Chromatin enrichment quantification for H2DBD-H1-Halo and Halo-H1DBD-H2 compared to full-length HSF1-Halo and Halo-HSF2 (data as shown in Figure 2E), Halo-H2DBD (data as shown in Supplementary Figure S6D, WT), and H1DBD-Halo (data as shown in Figure 5G). *n* = 30 cells for H2DBD-H1-Halo and 30 for Halo-H1DBD-H2 across three biological replicates.

We found that the HR-A/B region of HSF2–and to a lesser extent, HSF1–counteracts the intrinsic ability of the DBD to interact with mitotic DNA. This property contrasts sharply with SOX2 behavior where the DBD and NLS of SOX2 were shown to be the primary determinants for its mitotic chromatin association (10). Thus, to complement our studies with the HSFs, we investigated the importance of the DBD for mitotic chromatin coating within the SOX TF family. SOX2 and SOX13 share 55% DBD sequence similarity (Supplementary Figure S7C), but SOX2 is enriched on mitotic DNA (10,19) whereas SOX13 is excluded (19). We hypothesized that exchanging the DBDs between SOX2 and SOX13 could reverse their mitotic enrichment. We generated and expressed four HaloTagged TF constructs in WT JM8 cells: full-length Halo-SOX2 (as seen in previous figures) and Halo-SOX13, and two constructs with the DBDs exchanged (Figure 7A and B). As described previously (10,19), Halo-SOX2 is strongly enriched on mitotic chromatin while Halo-SOX13 is clearly excluded (Figure 7C and D). When the SOX2 DBD is replaced with that of SOX13 (Halo-S2S13DBD), the construct is evicted from mitotic DNA (Figure 7C and D). Accordingly, when the SOX13 DBD is replaced with that of SOX2 (Halo-S13S2DBD), the construct coats to a similar degree as full-length Halo-SOX2 (Figure 7C and D). Thus, the SOX2 DBD is both necessary and sufficient to induce mitotic chromatin coating.

We next asked whether either HSF DBD is sufficient to induce the mitotic coating of the other HSF. To address this question, we exchanged the DBDs between HSF1 and HSF2, generating two domain-swapped constructs: H2DBD-H1-Halo, in which the DBD of HSF1 is replaced with the HSF2 DBD, and Halo-H1DBD-H2, in which the DBD of HSF2 is replaced with the HSF1 DBD (Figure 7E). Each construct was overexpressed in WT JM8 cells (Figure 7F). Live-cell imaging of both chimeric constructs revealed clear exclusion from mitotic DNA, with no significant difference in chromatin enrichment from the full-length HSF constructs (Figure 7G and H). Though replacement of the HSF1 DBD with HSF2 DBD slightly increased enrichment, this effect is moderate in comparison to the effects of removing HR-A/B from HSF2 DBD (Figure 5D and E). Additionally, both H2DBD-H1-Halo and Halo-H1DBD-H2 are still clearly excluded, and significantly less enriched than their respective DBDs in isolation (Figure 7H). Therefore, neither DBD is sufficient to induce mitotic coating of the other HSF. This data further suggests that the non-DBD domains of both HSF1 and HSF2 promote exclusion from mitotic DNA, and that these domains affect both the HSF1 and HSF2 DBDs interchangeably. Thus, while the mitotic behavior of TFs like SOX2 and SOX13 is driven by the DBD, exclusion of HSF1-Halo and Halo-HSF2 from mitotic DNA is directed by non-DBD domains.

## DISCUSSION

In this paper, we investigate the mitotic DNA interactions of two related TFs: HSF1 and HSF2. Though the DBDs of these TFs are highly conserved, the two HSFs maintain different degrees of site-specific binding to mitotic DNA in mESCs. Despite this difference in specific binding, imaging analyses show surprisingly similar behaviors when these factors interact with mitotic DNA. Neither HaloTagged HSF1 nor HSF2 coat mitotic chromosomes as seen with live-cell imaging, and both HSF1-Halo and Halo-HSF2 interact with mitotic DNA more dynamically than with interphase chromatin, and these behaviors are not influenced by the nuclear import or export pathways. Instead, we found that the non-DBD domains of the HSFs not only contribute to increased dynamics but also dictate their exclusion from mitotic DNA.

We have shown that, at least for the HSFs, site-specific binding and global mitotic coating are distinct abilities that can be measured with different methods. Currently, studies using live-cell imaging approaches have shown that many TFs coat mitotic DNA (10,15–17,19), but precisely how coating translates to specific binding has been in question (12). This uncertainty is largely because the methods used to measure specific binding, such as ChIP-seq, are reliant on fixation that alters DNA-TF interactions in mitosis (10). In some cases, coating of TFs like ESRRB (12,17) and SOX2 (Figures 1 and 2) on mitotic DNA corresponded to site-specific binding. However, some evidence also suggests that the coating of mitotic DNA by TFs does not directly relate to specific DNA-binding events. For example, a mutant version of FOXA1 that cannot recognize and bind to specific target sites was largely retained on mitotic chromosomes as visualized by live imaging, despite the loss of binding on specific loci (16). Therefore, previous studies have shown three modes of TF-mitotic DNA interactions: coating with site-specific binding, coating without site-specific binding, and neither coating nor binding site-specifically. Our study reveals a fourth mode: HSF1 and HSF2 cannot coat mitotic chromosomes but can bind specifically to target loci (Figures 1 and 2), albeit to varying degrees.

Our study does have its caveats. For one, because the CUT&Tag protocol relies on the protein-A-Tn5 fusion, we cannot completely rule out the possibility that the Tn5 fusion protein is preferentially binding to open chromatin, though this protocol has been optimized to reduce such preference (32,38). Second, in order to image the HSFs, we overexpressed HaloTagged HSF fusion proteins, which could behave differently from the endogenous protein. We have shown several lines of evidence to support that such a discrepancy is not occurring. First, the HaloTagged HSFs behaved similarly in WT and corresponding HSF KO cells (Figure 2 and Supplementary Figure S4C, D). Second, the HaloTagged HSFs behaved differently from the Halo-DBD only constructs, suggesting that the HaloTag is not causing exclusion of the full-length protein. Lastly, the CUT&Tag analysis of Halo-HSF2 shows that, although the signal is weaker than the endogenous, the tagged protein can bind site-specifically (Supplementary Figure S8). Furthermore, HSF2 DBD alone can bind site-specifically, even though its coating behavior is different from the full length HSF2. These data suggest that the HSFs exhibit a distinct mode of mitotic DNA interaction.

Despite differences in coating behavior, all of the constructs we examined interacted with DNA more dynamically in mitosis than in interphase (Figures 3A–C and 6B–D). This consistent pattern has also been shown for various other TFs using SPT or fluorescence recovery after photobleaching, including SOX2 (10), FOXA1 (16,19), CDX2 (19), HMGB2 (19), POU5F1 (19), TEAD1 (19) and others. The mechanisms underlying these increased dynamics for TFs during mitosis are not fully understood. It is possible that the drastic changes to the cellular landscape during mitosis contributes to these increased DNA-TF dynamics. For instance, chromosomes are globally reorganized and condensed during mitosis, which may affect the association rates and target search mechanism used by TFs (59–62). Another global change in mitosis is the dissolution of the nuclear membrane, which increases the space in which a TF can diffuse and may also impact association rates and target search. Furthermore, binding of many TFs is stabilized by cooperativity with other proteins (63–66), and loss of these protein-protein interactions during mitosis, due to the global and massive decrease in transcriptional activity, may also contribute to the increased *k*_off_ (Supplementary Figures S5C and S7B). Surprisingly, our SPT data for the full-length versus DBD truncation of HSF2 suggests that at least part of the DNA-TF dynamics may be inherent to specific TF domains. The relative residence times for Halo-HSF2 in mitosis is 42.5% relative to interphase and is increased to 68.3% for Halo-H2DBD (Figures 3E, and 6F), indicating that on average the DBD-only molecules interact with mitotic DNA longer than full-length HSF2. The domains removed from the HSF2 DBD truncation include the trimerization domains (HR-A/B and C), the regulatory domain, and the transactivation domain; how these domains specifically contribute to increased mitotic DNA-TF interaction dynamics remains to be seen.

Previous studies suggested that the DBD is one of the major determinants of mitotic DNA coating (10,26,67). Indeed, SOX2 DBD alone is able to coat mitotic DNA (10), and our results show that the SOX2 DBD can induce mitotic coating of SOX13 (Figure 7C and D). However, at least in the case of the HSFs, we show that the DBD is not the dominant driver of mitotic DNA interactions. In fact, even though the HSF2 DBD—and to a lesser extent, the HSF1 DBD—can coat mitotic chromosomes in isolation, neither DBD is able to induce mitotic DNA coating of its full-length protein or of the non-DBD domains of the complementary HSF. Therefore, the non-DBD regions of the HSFs, perhaps the trimerization domain, not only contribute to DNA-TF interaction dynamics, but ultimately drive the mode of HSF behavior during mitosis (i.e. not coating but binding site-specifically). Both HSF1 and HSF2 generally bind to DNA as trimers (68,69), which form a stable DNA-enveloping structure (70,71). In vitro studies have revealed that monomeric HSF1 is able to bind DNA, albeit at lower affinity than trimerized protein (72). The equilibrium dissociation constant (*K*_D_) of monomeric HSF1 was ∼10 times higher than that of the trimer, whereas trimerization deficient HSF1 with the HR domains excised had ∼70 times higher *K*_D_. Our SPT results show that the Halo-H2DBD, which also lacks the trimerization HR domains, has only slightly lower *k*_off_ rates (longer residence times) on mitotic chromosomes compared to the full length HSF2 (Supplementary Figure S7B). Assuming that the binding affinity of HSF2 trimers is similar to that of HSF1, and that *K*_D_ = *k*_off_/*k*_on_ (73), *k*_on_ for the truncated H2DBD protein would be at least 20× decreased compared to the full length HSF2. The decreased *k*_on_ means a much slower rate of finding specific target sites, which could signify increased time spent in diffusion and/or target search and non-specific DNA associations (74). The change in coating behavior that we observed for Halo-H2DBD (Figure 5D and E) would be consistent with increased non-specific DNA associations, without effects on site-specific binding (Figure 6G–I). Taken together, our findings highlight the importance of multimerization domains like the HR in mitotic DNA association of the HSFs. Since many TFs act as dimers or trimers, such multimerization capacity could determine the mode of TF-mitotic DNA interactions for these TFs.

## DATA AND AVAILABILITY

The CUT&Tag datasets generated in this study have been deposited to Gene Expression Omnibus (https://www.ncbi.nlm.nih.gov/geo/) and are available as raw and processed files through accession numbers GSE214219. Plasmid constructs are deposited on Addgene.

## Supplementary Material

gkad304_Supplemental_Files
