# Supplementary material for: Heat shock transcription factors demonstrate a distinct mode of interaction with mitotic chromosomes
Source: Nucleic Acids Res. 2023 Apr 28;51(10):5040–55. doi: 10.1093/nar/gkad304 (PMC10250243; doi:10.1093/nar/gkad304)
Supplement: gkad304_Supplemental_Files [file gkad304_supplemental_files.zip › Resubmission2_Supplementary.pdf]

Supplementary Data

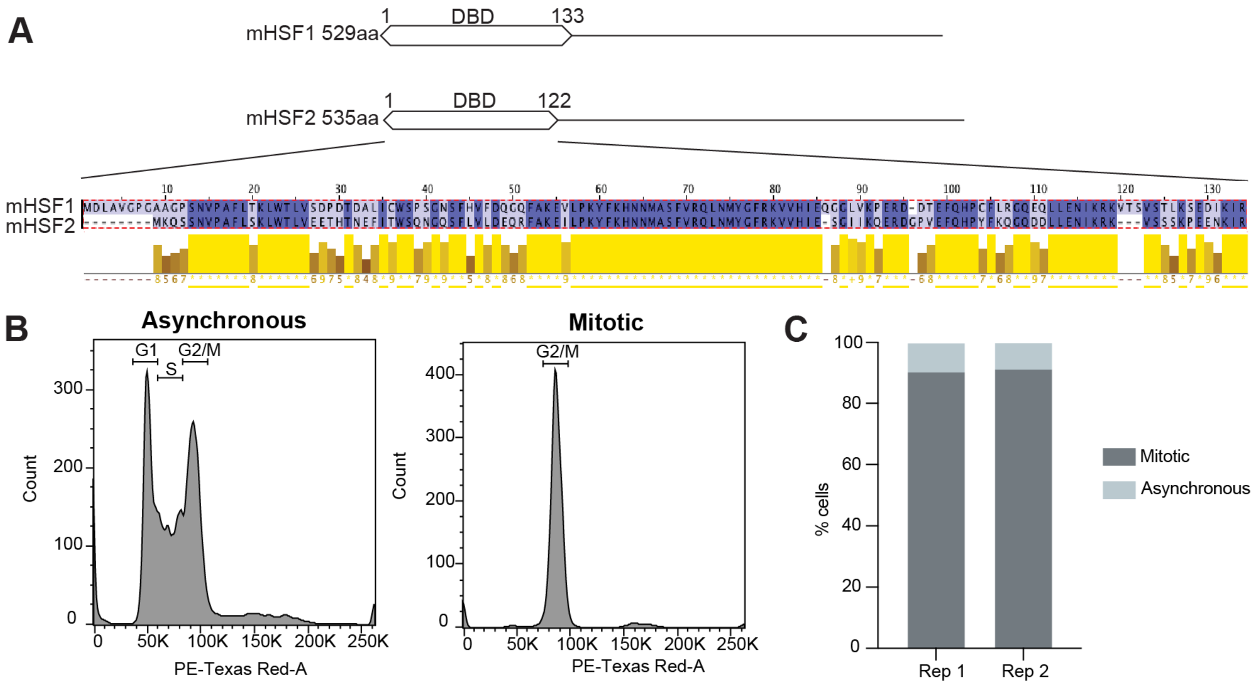

Supplemental Figure 1: HSF DBD sequence alignment, and efficiency of mitotic enrichment

(A) HSF1 DBD and HSF2 DBD sequence alignment (blue) and scoring matrices (yellow). Alignment produced with Jalview. **(B)** Representative histograms of asynchronous and mitotic cells based on the DNA content. **(C)** Mitotic indexes of biological replicates used as mitotic fractions for CUT&Tag assay.

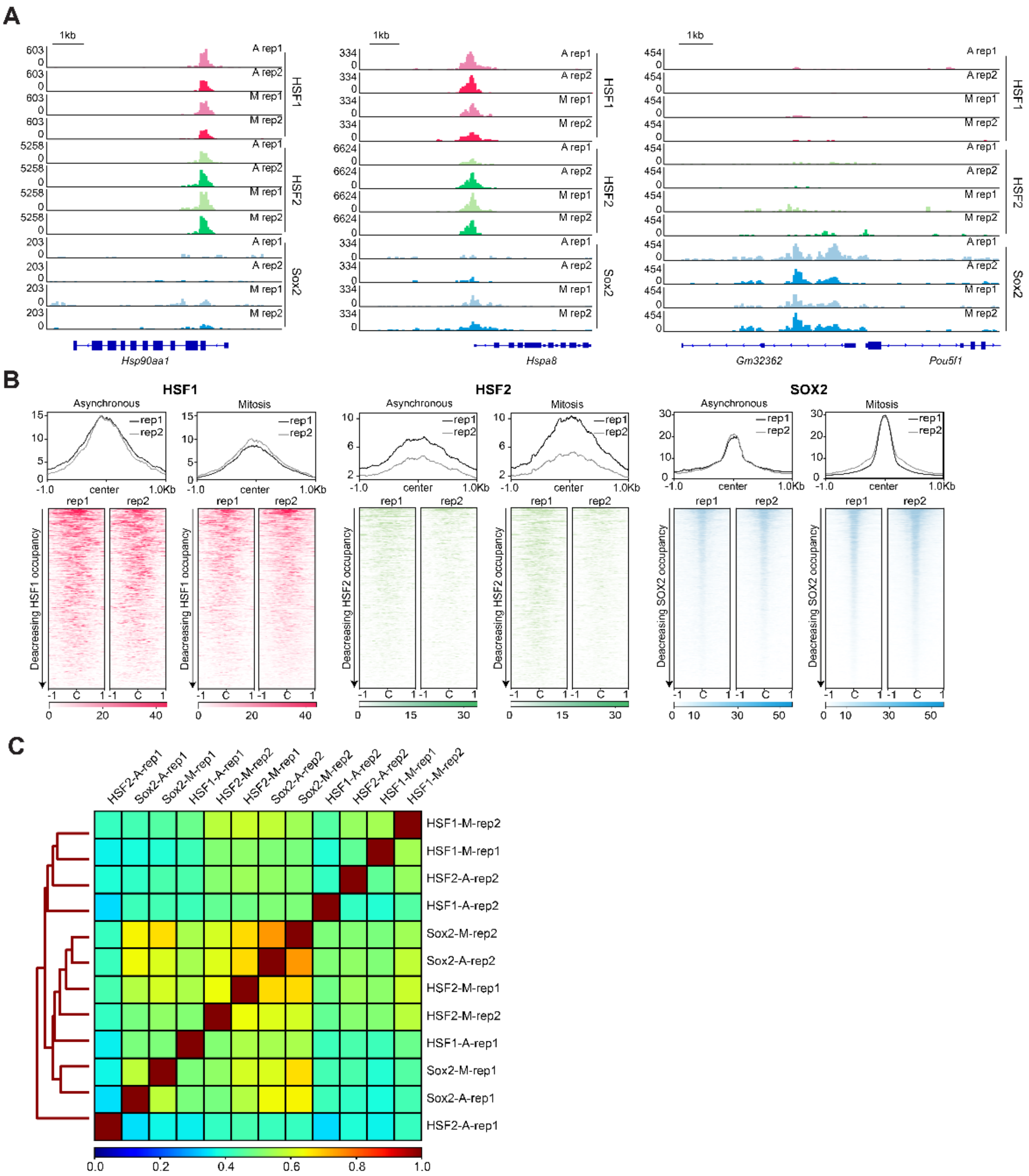

Supplemental Figure 2: Replicate analysis of CUT&Tag data

(A) Gene browser tracks of biological replicates for HSF1, HSF2, and SOX2 over *Hsp90aa1, Hspa8, and Pou5f1* loci in asynchronous (A) and mitotic (M) cells. Please note that the scale for HSF2 at *Hsp90aa1* and *Hspa8* is different from the scale for HSF1 and SOX2. **(B)** Genome-wide average plots (top) and heatmaps (bottom) of biological replicates of HSF1 (left), HSF2 (middle), and SOX2 (right) CUT&Tag in asynchronous (A) and mitotic (M) cells. CUT&Tag signal was calculated in a 2 kb window surrounding binding sites of the respective TF. For

heatmaps, binding sites were ordered by decreasing occupancy of the given TF. **(C)** Pearson correlation analysis of HSF1, HSF2, and SOX2 biological replicates in asynchronous (A) and mitotic (M) cells.

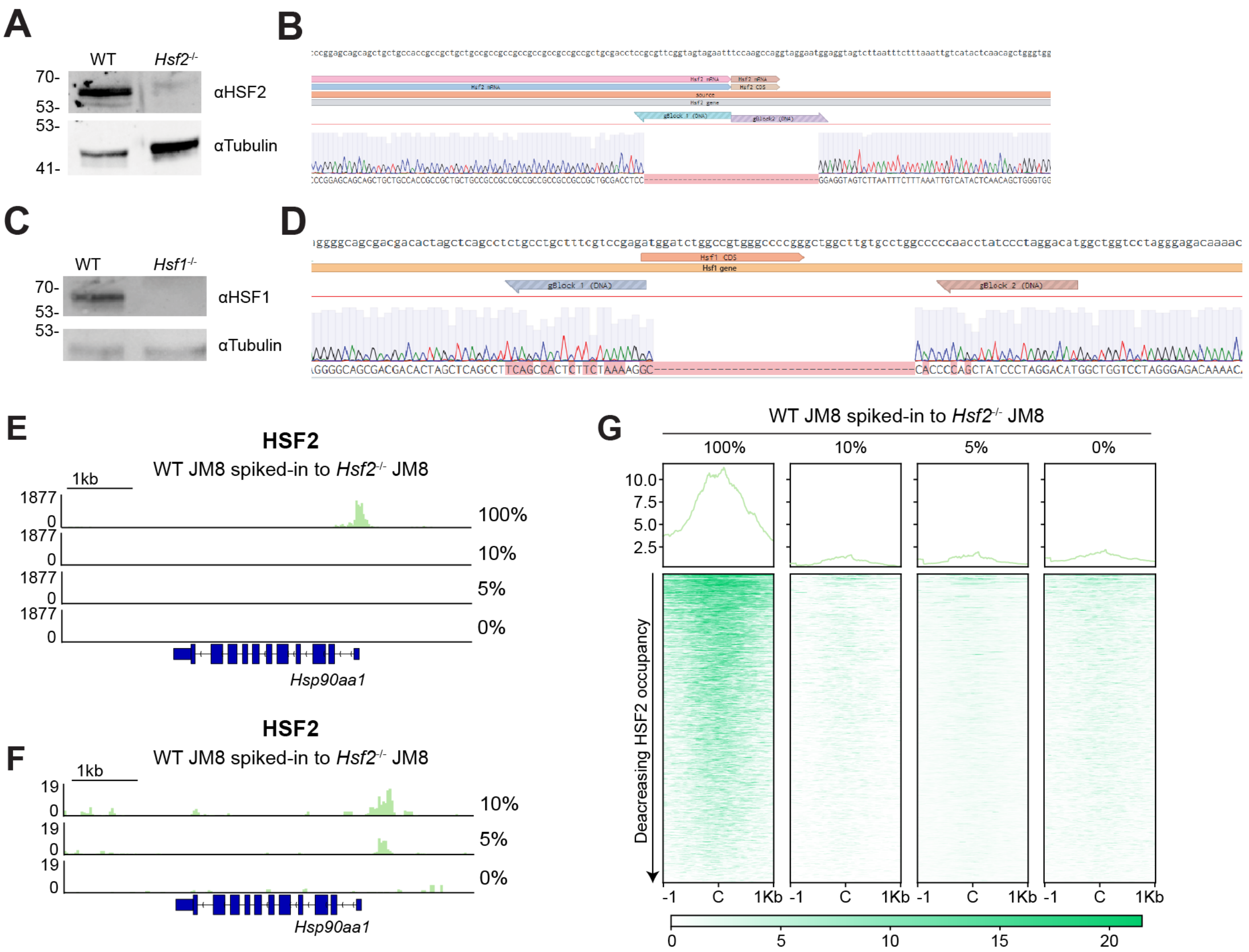

Supplemental Figure 3: HSF knock-out verification, and CUT&Tag cellular contamination =spike-in assay

(A) Western blot of WT and *Hsf2*$^{-/-}$ JM8 cells using α-HSF2. Tubulin is shown as a loading control. **(B)** Sanger sequencing trace showing the region of *Hsf2* gene deleted in *Hsf2*$^{-/-}$ JM8 cells. **(C)** Western blot of WT and *Hsf1*$^{-/-}$ JM8 cells using α-HSF1. Tubulin is shown as a loading control. **(D)** Sanger sequencing trace showing the region of *Hsf1* gene deleted in *Hsf1*$^{-/-}$ JM8 cells. **(E)** Gene browser tracks of HSF2 over the *Hsp90aa1* locus. Indicated percentages of WT JM8 were spiked-in to *Hsf2*$^{-/-}$ JM8 to show that up to 10% cellular contamination does not drastically impact CUT&Tag signal. **(F)** Data as in (E) without 100% sample, with the scale zoomed in. **(G)** Genome-wide average plots (top) and heatmaps (bottom) of HSF2. Indicated percentages of WT JM8 were spiked-in to *Hsf2*$^{-/-}$ JM8 to show that up to 10% cellular contamination does not drastically impact CUT&Tag signal. CUT&Tag signal was calculated in a 2 kb window surrounding HSF2 binding sites. For heatmaps, binding sites were ordered by decreasing HSF2 occupancy. Data averaged across two biological replicates.

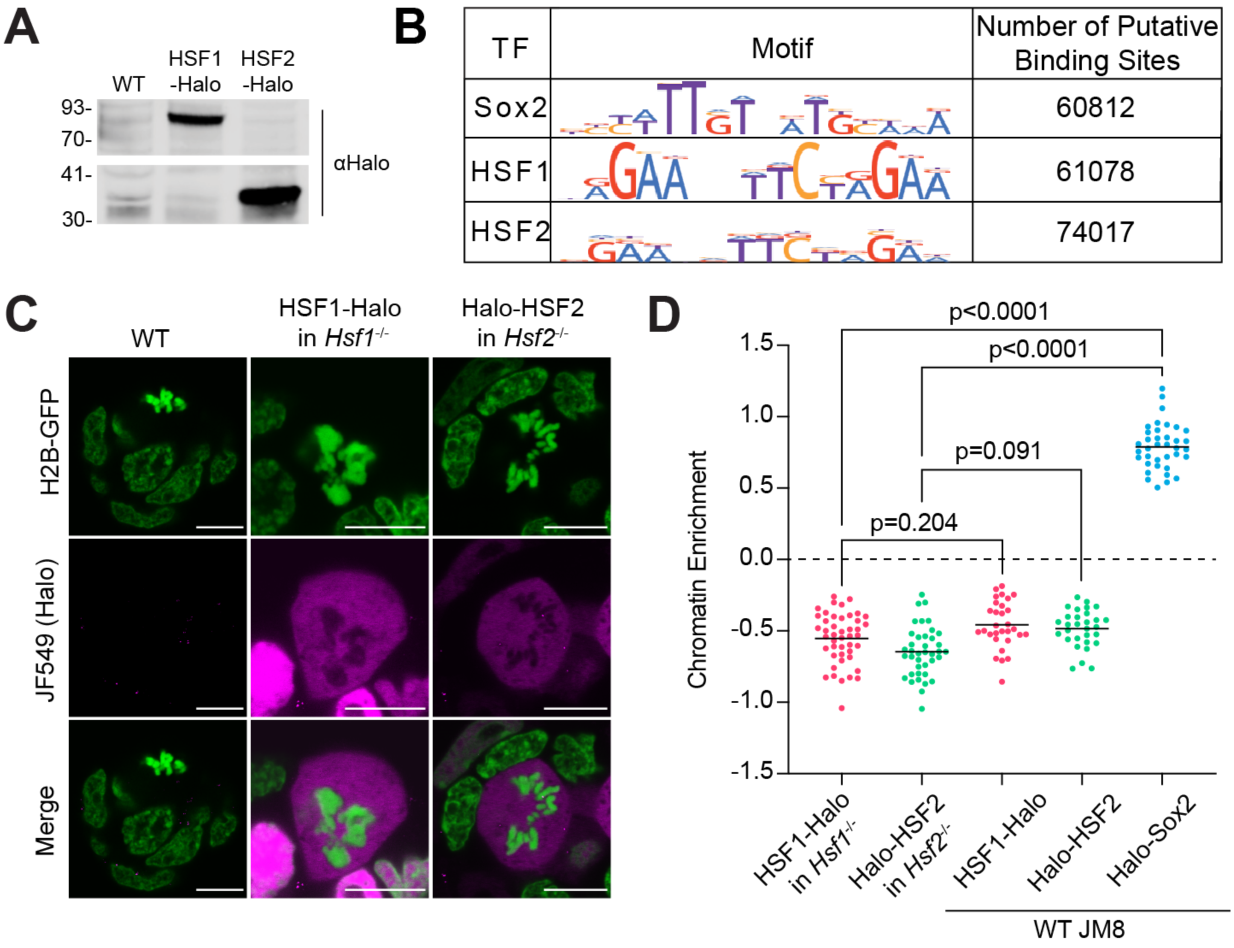

Supplementary Figure 4: C-terminal HaloTagging of HSF2, putative TF binding sites, and HaloTagged HSF imaging in HSF KO background

(A) Western blot of overexpressed HSF1-Halo and HSF2-Halo in JM8 cells using α-Halo. C-terminal tagging of HSF2 resulted in truncation of the protein. **(B)** Number of putative binding sites for SOX2, HSF1, and HSF2 throughout the mouse genome. Binding motifs used for search of potential binding sites are shown for each TF. **(C)** Live-cell fluorescent imaging of HSF1-Halo and Halo-HSF2 (magenta) labeled with 200 nM JF549 dye in *Hsf1*$^{-/-}$ and *Hsf2*$^{-/-}$ cells, respectively. DNA is visualized with H2B-GFP overexpression (green). Scale bars represent 10 µm. **(D)** Chromatin enrichment quantification comparing the HaloTagged HSFs in *Hsf1*$^{-/-}$ and *Hsf2*$^{-/-}$ cells (n=45 cells for HSF1-Halo and 39 for Halo-HSF2 across 3 biological replicates) and in WT JM8 (data as seen in Fig. 2E). Data are visualized as individual data points with mean value indicated.

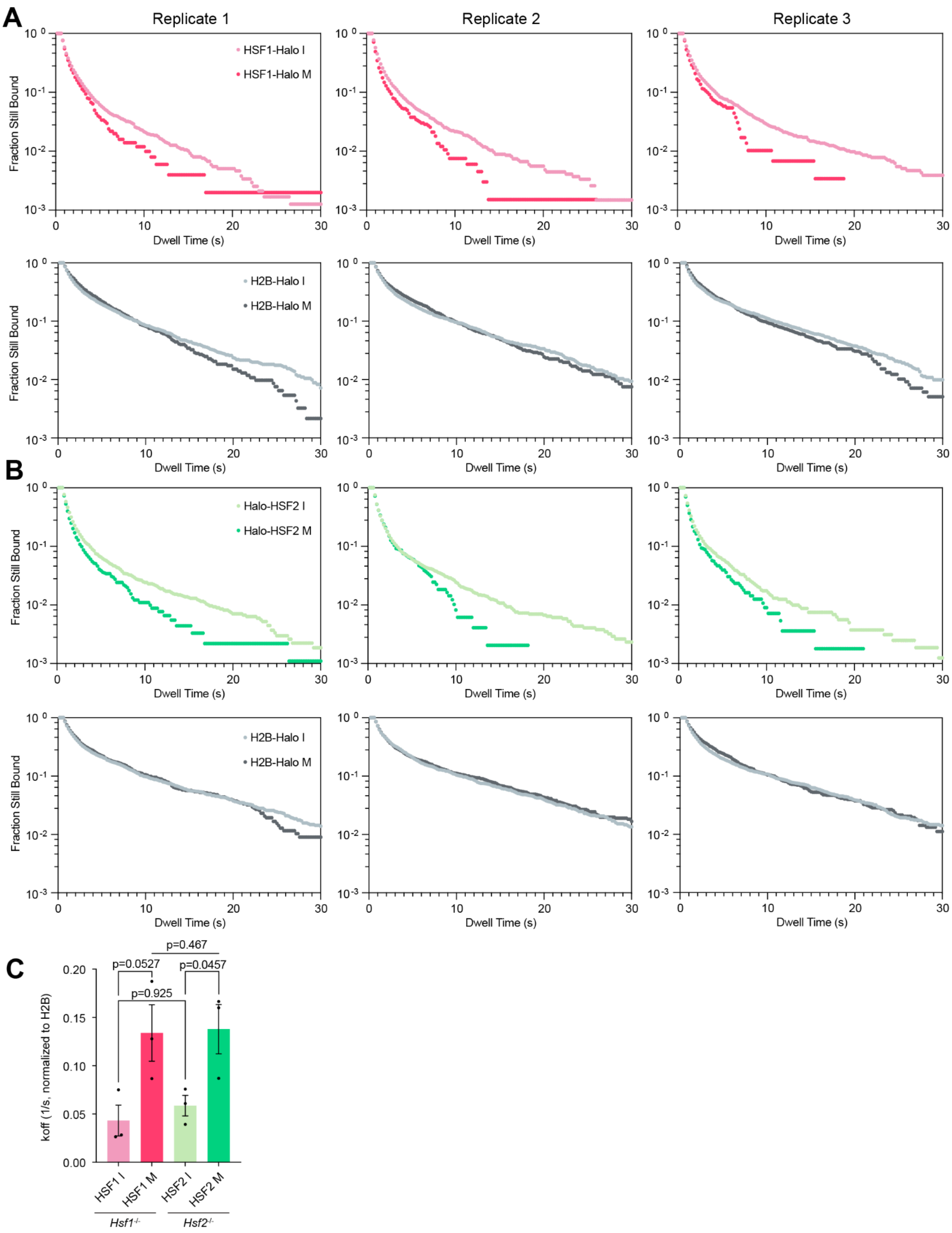

Supplemental Figure 5: Replicate dwell curves for HSF1-Halo and Halo-HSF2 SPT
(A) Decay curves for each biological replicate of HSF1-Halo in *Hsf1*$^{-/-}$ (red, top) and the associated H2B-Halo replicate (gray, bottom) representing the fraction of HaloTagged molecules

bound to DNA in interphase (I, lighter color) and mitosis (M, darker color) over time. Technical replicates: n=19 cells for H2B-Halo I Rep1, 11 for H2B-Halo I Rep2, 10 for H2B-Halo I Rep3, 9 for H2B-Halo M Rep1, 11 for H2B-Halo M Rep2, 10 for H2B-Halo M Rep3, 12 for HSF1-Halo I Rep1, 12 for HSF1-Halo I Rep2, 10 for HSF1-Halo I Rep3, 5 for HSF1-Halo M Rep1, 10 for HSF1-Halo M Rep2, and 8 for HSF1-Halo M Rep3. **(B)** Decay curves for each biological replicate of Halo-HSF2 in *Hsf2*$^{-/-}$ (green, top) and the associated H2B-Halo replicate (gray, bottom) representing the fraction of HaloTagged molecules bound to DNA in interphase (I, lighter color) and mitosis (M, darker color) over time. Technical replicates: n=12 cells for H2B-Halo I Rep1, 9 for H2B-Halo I Rep2, 10 for H2B-Halo I Rep3, 9 for H2B-Halo M Rep1, 11 for H2B-Halo M Rep2, 10 for H2B-Halo M Rep3, 11 for Halo-HSF2 I Rep1, 12 for Halo-HSF2 I Rep2, 9 for Halo-HSF2 I Rep3, 11 for Halo-HSF2 M Rep1, 9 for Halo-HSF2 M Rep2, and 9 for Halo-HSF2 M Rep3. **(C)** H2B-corrected k_{off} values for HSF1-Halo and Halo-HSF2 constructs in interphase and mitosis, where each dot represents one biological replicate. Corrected k_{off} = k_{off}(TF) - k_{off}(H2B). Data depicted as mean ± SEM.

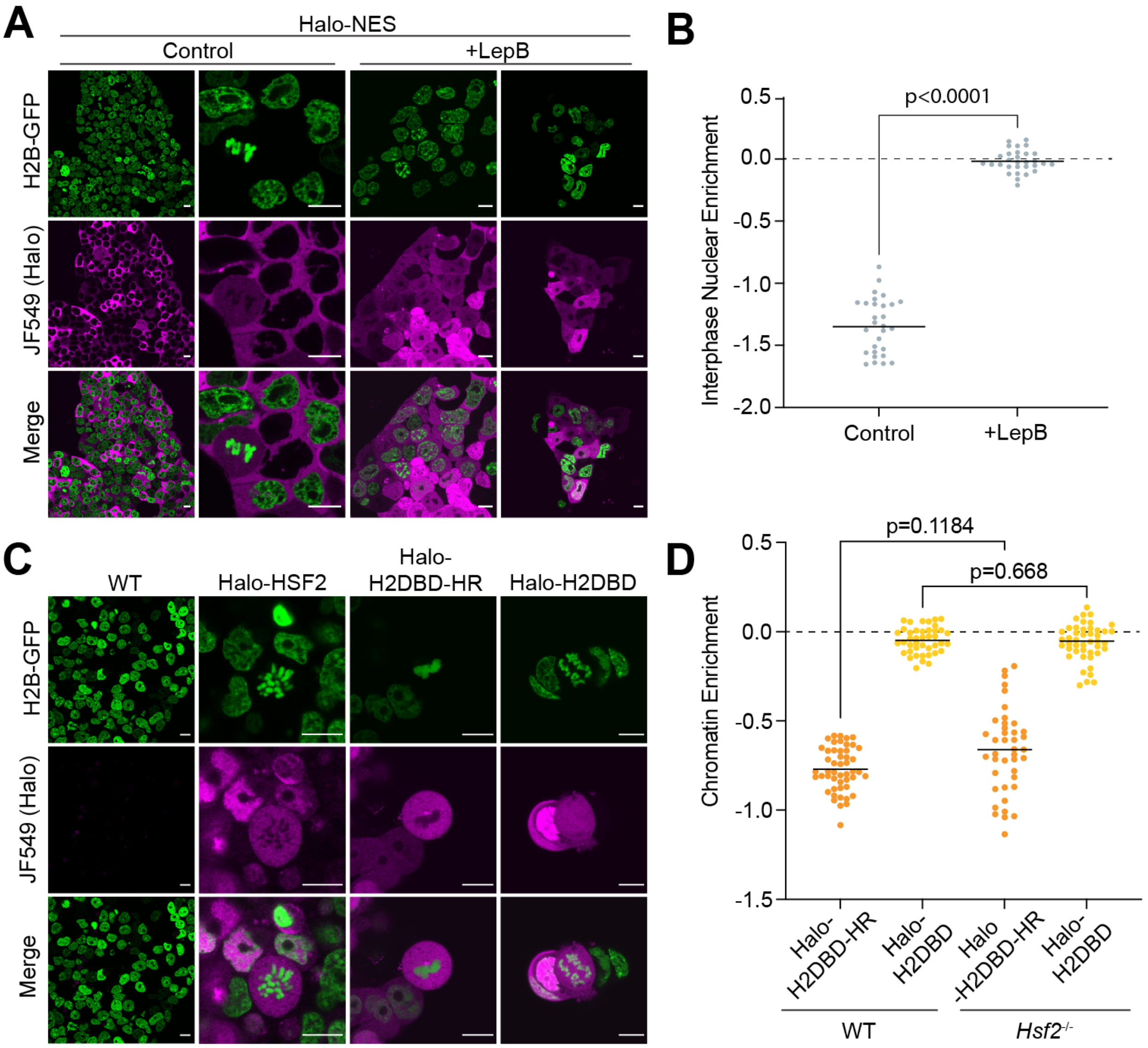

Supplemental Figure 6: Validation of nuclear export inhibition, and HSF2 truncations in WT JM8 versus in HSF2 KO cells

(A) Live-cell imaging of WT JM8 cells labeled with 200 nM JF549 dye (magenta) expressing Halo-NES construct. DNA is visualized by H2B-GFP overexpression (green). Scale bars are 10µm. Cells were either treated with 10 ng/mL leptomycin B (LepB) (+LepB) or with vehicle (Control) for 1h before imaging. **(B)** Nuclear enrichment quantification for the Halo-NES constructs (n=29 cells for NES-Halo control and 30 for NES-Halo +LepB across 3 biological replicates) in interphase cells. Data are visualized as individual data points with mean value indicated. **(C)** Live-cell imaging of WT JM8 cells expressing Halo-HSF2 and HSF2 truncation constructs labeled with 200 nM JF549 dye (magenta). DNA is visualized by H2B-GFP overexpression (green). Scale bars are 10µm. **(D)** Chromatin enrichment quantification comparing the HSF2 truncation constructs in WT JM8 cells (n=49 cells for Halo-H2DBD-HR and 42 for Halo-H2DBD across 3 biological replicates) and in *Hsf2*$^{-/-}$ cells (data as in Fig. 5E).

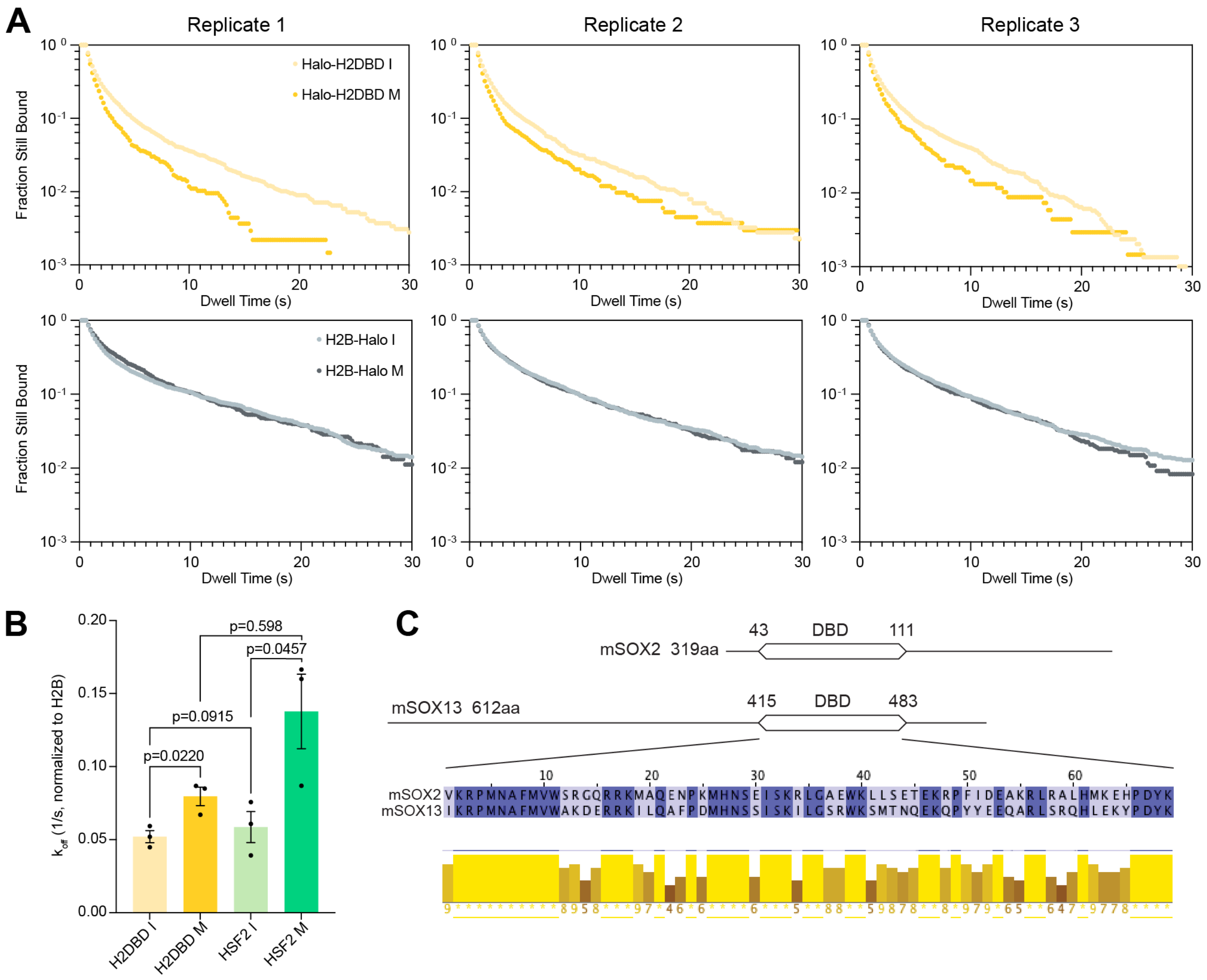

Supplementary Figure 7: Replicate dwell curves for Halo-H2DBD SPT, and SOX DBD sequence alignment

(A) Decay curves for each biological replicate of Halo-H2DBD (yellow, top) and the associated H2B-Halo replicate (gray, bottom) representing the fraction of HaloTagged molecules bound to DNA in interphase (I, lighter color) and mitosis (M, darker color) over time. Technical replicates: n=10 cells for H2B-Halo I Rep1, 10 for H2B-Halo I Rep2, 11 for H2B-Halo I Rep3, 10 for H2B-Halo M Rep1, 11 for H2B-Halo M Rep2, 12 for H2B-Halo M Rep3, 12 for Halo-H2DBD I Rep1, 11 for Halo-H2DBD I Rep2, 11 for Halo-H2DBD I Rep3, 11 for Halo-H2DBD M Rep1, 10 for Halo-H2DBD M Rep2, and 8 for Halo-H2DBD M Rep3. **(B)** H2B-corrected k_{off} values for Halo-HSF2 (data as seen in Fig. S5C) and Halo-H2DBD constructs in interphase and mitosis, where each dot represents one biological replicate. Corrected $k_{\mathrm{off}} = k_{\mathrm{off}}(\mathrm{TF}) - k_{\mathrm{off}}(\mathrm{H2B})$. Data depicted as mean ± SEM. **(C)** SOX2 DBD and SOX13 DBD sequence alignment (blue) and scoring matrices (yellow). Alignment produced with Jalview.

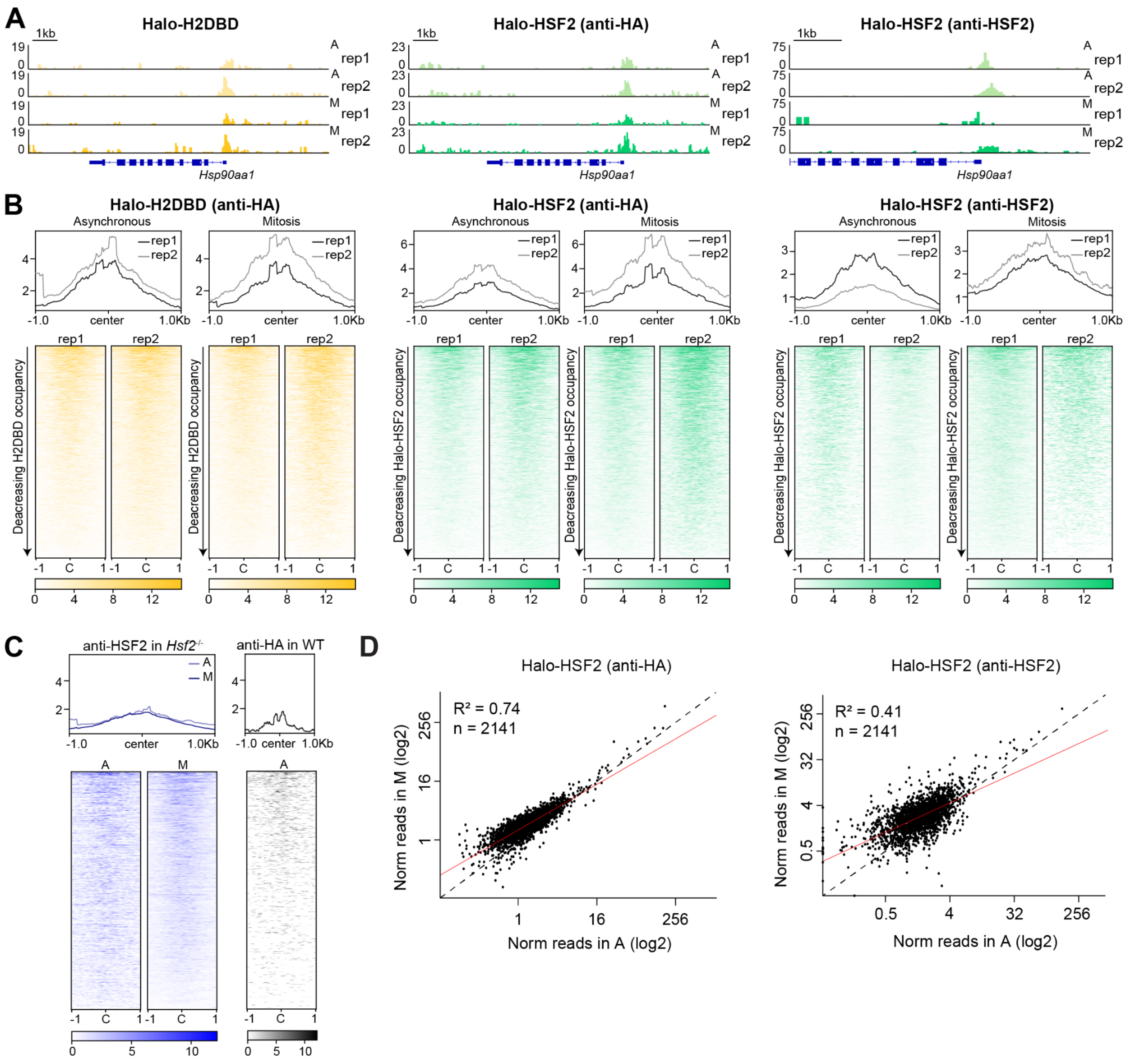

Supplemental Figure 8: Replicate analysis of Halo-H2DBD and Halo-HSF2 CUT&Tag
(A) Gene browser tracks of biological replicates for Halo-H2DBD (left), HA-tagged Halo-HSF2 using α-HA (middle), and Halo-HSF2 using α-HSF2 (right) over *Hsp90aa1* locus in asynchronous (A) and mitotic (M) *Hsf2*$^{-/-}$ cells. **(B)** Genome-wide average plots (top) and heatmaps (bottom) of biological replicates of Halo-H2DBD (left), HA-tagged Halo-HSF2 using α-HA (middle) and Halo-HSF2 using α-HSF2 (right) CUT&Tag in asynchronous (A) and mitotic (M) *Hsf2*$^{-/-}$ cells. CUT&Tag signal was calculated in a 2 kb window surrounding HSF2 binding sites. For heatmaps, binding sites were ordered by decreasing Halo-H2DBD occupancy. **(C)** Genome-wide average plots (top) and heatmaps (bottom) of α-HSF2 CUT&Tag in *Hsf2*$^{-/-}$ cells in asynchronous (A) and mitotic (M) cells (left) and α-HA control in WT JM8 cells (right). CUT&Tag signal was calculated in a 2 kb window surrounding HSF2 binding sites. **(D)** Normalized read counts of Halo-HSF2 using α-HA (left) or α-HSF2 (right) for each binding site in asynchronous (A) and mitotic (M) cells displayed as a scatterplot. N denotes number of analyzed sites.

Supplementary Video 1: SPT for residence time analysis of H2B-Halo overexpression cells in interphase. Related to Figure 3. Imaging H2B-Halo molecules in interphase ES cells at 5 Hz. Movie fps = 10.

Supplementary Video 2: SPT for residence time analysis of H2B-Halo overexpression cells in mitosis. Related to Figure 3. Imaging H2B-Halo molecules in mitotic ES cells at 5 Hz. Movie fps = 10.

Supplementary Video 3: SPT for residence time analysis of Halo-HSF2 overexpression cells in interphase. Related to Figure 3. Imaging Halo-HSF2 molecules in interphase *Hsf2*$^{-/-}$ ES cells at 5 Hz. Movie fps = 10.

Supplementary Video 4: SPT for residence time analysis of Halo-HSF2 overexpression cells in mitosis. Related to Figure 3. Imaging H2B-Halo molecules in mitotic *Hsf2*$^{-/-}$ ES cells at 5 Hz. Movie fps = 10.

Supplementary Video 5: SPT for residence time analysis of Halo-H2DBD overexpression cells in interphase. Related to Figure 6. Imaging Halo-H2DBD molecules in interphase ES cells at 5 Hz. Movie fps = 10.

Supplementary Video 6: SPT for residence time analysis of Halo-H2DBD overexpression cells in mitosis. Related to Figure 6. Imaging Halo-H2DBD molecules in mitotic ES cells at 5 Hz. Movie fps = 10.

Supplementary Table 1: Constructs and primers

Construct	Amplicon Name	Fwd Primer (5' -> 3')	Rev Primer (5' -> 3')
Halo	from Teves S.S. 2016		
Halo-HSF2	HSF2	GATAACTCTAGAATGAAGCAGAGTTCCAACGTGCC	AGGGGCGCGGCCGCTTAACTATCTAAAAGCGGCATATCACTATCCAGAG G
HSF1-Halo	from Teves S.S. 2016		
H2DBD-H1-Halo	HSF2 DBD	TGACCGGCGCCTACTATGAAGCAGAGTTCCAAC	CACTGTCCTGACGAATTTTATTTTCCTCTGG
H2DBD-H1-Halo	HSF1 without DBD	AAATTCGTCAGGACAGTGTCACCCGG	GACTAGTCCTCGAGGAGGAGACAGTGGGGTCCTTG
Halo-Sox2	from Teves S.S. 2016		
Halo-Sox13	Sox13 cDNA	TTTCAGAGCGATAACTCTAGGTCCATGCAGAGCCCCGT	GGAGGGAGAGGGGCGCGGCCTCAGTCTGTGAGTACCACCAGC
Halo-S2S13DBD	Sox13 DBD	AGAAGAACAGCCCGGACCGCATCAAGAGACCCATGAATGC	GTTTTCCGCCGCGGCCGGTACTTATAGTCAGGGTATTTCTCCA
Halo-S13S2DBD	Sox2 DBD	AGTCTCGGAACAGCAGCCACGTCAAGAGGCCCATGAACGC	CGCTTAGGCCTTGGCTTGTATTTATAATCCGGGTGCTCCTTC
NLS-Halo	gBlock	CACGGTAACCCGACCTCCTCCTACGTGTGGCGCAACATC	GCCCGGTCTCGACCTGAGCTTTAAACTTACCTAGACGGCGGAC
NLS-Halo-HSF2	gBlock	CACGGTAACCCGACCTCCTCCTACGTGTGGCGCAACATC	GCCCGGTCTCGACCTGAGCTTTAAACTTACCTAGACGGCGGAC
Halo-H2DBD-HR		ACACAAATGGAGCCCCAAAGTAAGCCCCTCTCCCTCCCCCCC C	GGGGGGGGAGGGAGAGGGGCTTACTTTGGGGCTCCATTTGTGT
Halo-H2DBD	Set 1	AGAACATTAAAAGGAAGGTTTAAGCCCCTCTCCCTCCCCCCCC	GGGGGGGGAGGGAGAGGGGCTTAAACCTTCCTTTTAATGTTCTCC
Halo-H2DBD	Set 2	CAGAGGAAAATAAAATTCGTGGTGGCGGTGGCTCGAAACGTA AAAGGCCTCTACT	AGTAGAGGCCTTTTACGTTTCGAGCCACCGCCACCACGAATTTTATTTTC CTCTGGT
NES-Halo	gBlock	CACGGTAACCCGACCTCCTCCTACGTGTGGCGCAACATC	GCCCGGTCTCGACCTGAGCTTTAAACTTACCTAGACGGCGGAC
HA-Halo-HSF2	gBlock	CACGGTAACCCGACCTCCTCCTACGTGTGGCGCAACATC	CAGAGGTTGATTGTCGACTCAGAAGAACTCGTCAAGAAGG
H1DBD-Halo-HA	Set 1	CAGCACACTCTGTGCCCAAGAATTCCTCGAGGACTAGTCC	TCCAGAGGTTGATTGTCGACCAGAGGTTGATTGTCGACTCAGAAGAAC TCG
H1DBD-Halo-HA	Set 2	GAGTCGACAATCAACCTCTGGTCGACAATCAACCTCTGGA	ATCAGAGGGATCTTTCTCTTCGAGCCACCGCCACCGCGTATTTTTATGTC CTCACT
H1DBD-Halo-HA	NLS2	GTGAGGACATAAAAATACGCGGTGGCGGTGGCTCGAAGAGA AAGATCCCTCTGATGTTG	GGACTAGTCCTCGAGGAATTCTTGGGCACAGAGTGTGCTG
H1DBD-Halo-HA	H1DBD-Halo with HA	AGCTGTGACCGGCGCCTACTATGGATCTGGCCGTG	AGGGGCGCGGCCTTAAGCGTAATCTGGAACATCGTATGGGTACATCTTG TCGTCATCGTC
H1DBD-HR-Halo	Set 1	CAGCACACTCTGTGCCCAAGAATTCCTCGAGGACTAGTCC	CAGAGGTTGATTGTCGACTCAGAAGAACTCGTCAAGAAGG
H1DBD-HR-Halo	Set 2	GAGTCGACAATCAACCTCTGAATTTGTGAAAGATTGACTGGT	GGACTAGTCCTCGAGGAATTCTTGGGCACAGAGTGTGCTG
Halo-H1DBD-H2	HSF1 DBD	TGACCGGCGCCTACTATGGATCTGGCCGTGGGC	TTTTTGTTAAATCTTCCTGGCGTATTTTTATGTCCTCACTCTTCAGG
Halo-H1DBD-H2	HSF2 without DBD	CAGGAAGATTTAACAAAAATTATTAG	GACTAGTCCTCGAGGTCTTACTATCTAAAAGCGGC
Halo-H2DBD with HA tag	gBlock	CACGGTAACCCGACCTCCTCCTACGTGTGGCGCAACATC	CAGAGGTTGATTGTCGACTCAGAAGAACTCGTCAAGAAGG

Supplementary Table 2: CRISPR gRNA and screening primers

	gRNA 1	gRNA 2	Screening Primer 1 (Rev) (5' -> 3')	Screening Primer 2 (Fwd) (5' -> 3')	Screening Primer 3 (Rev) (5' -> 3')
HSF1 KO	TCTCGGACGAAAGC AGGCAG	ATGTCCTAGGGATAG GTTGG	AAAGTAGGCAGGGGAGATC ACGG	GGGCAGGGGGAAACATACA GGT	GAGATACACACAGTCCCCCCA GC
HSF2 KO	AATTCTACTACCGAA CGCGG	TCCAAGCCAGGTAGG AATGG	AGTCAACTGCCCTTGGGCT TCTC	CCCTGTTCAATAGCAGATGC AGAGTT	TAACATGCGAAACCATGTCTG ATTTACTCCC

Supplementary Table 3: CUT&Tag scaling values

		mm10				drosophila					
Samples	Total Reads	Aligned 1	Aligned>1	Total Mapped Reads	Alignment Rate	Aligned 1	Aligned>1	Total Mapped Reads	Alignment Rate	100000/ Drosophila Reads	Scaling Value
JM8-A-HSF-1_S50	13269819	9184086	2993062	12177148	91.77%	204944	65302	270246	2.04%	0.370033229	1
JM8-A-HSF-2_S51	8839754	5928199	2226788	8154987	92.25%	140498	49202	189700	2.15%	0.5271481286	1
JM8-M-H1-rep1_S74	7782193	5367142	1684396	7051538	90.61%	218269	57786	276055	3.55%	0.3622466537	0.9789570919
JM8-M-H1-rep2_S82	14260143	9590624	3276429	12867053	90.23%	344467	102853	447320	3.14%	0.2235536082	0.4240811947
JM8-A-H2-rep1_S71	7331874	5762643	1283074	7045717	96.10%	16657	10324	26981	0.48%	37.06311849	1
JM8-A-H2-rep2_S79	2803297	1851025	754203	2605228	92.93%	11420	11060	22480	0.90%	44.48398577	1
JM8-M-H2-rep1_S75	10391539	6912567	1913141	8825708	84.93%	7072	35633	42705	0.46%	23.41646177	0.6317995551
JM8-M-H2-rep2_S83	6501248	3642772	1351678	4994450	76.82%	7923	28410	36333	0.60%	27.52318829	0.6187212727
JM8-A-Sox2-rep1_S72	32658821	21656681	9600054	31256735	95.71%	27399	30308	57707	0.22%	17.32892023	1
JM8-A-Sox2-rep2_S80	18013185	11135678	5697825	16833503	93.45%	25784	25030	50814	0.33%	19.67961585	1
JM8-M-Sox2-rep1_S76	64461421	39307201	21880183	61187384	94.92%	59675	150247	209922	0.38%	4.763674127	0.2748973428
JM8-M-Sox2-rep2_S84	112266139	62676311	43724531	106400842	94.78%	101418	103907	205325	0.21%	4.87032753	0.2474808231
A-IgG-rep1_S26	2143027	1435233	514503	1949736	90.98%	6779	4513	11292	0.62%	88.55827134	
M-IgG-rep1_S31	231972	146012	69739	215751	93.01%	1580	731	2311	1.17%	432.7131112	
M-IgG-rep2_S47	452941	305848	122683	428531	94.61%	3823	2472	6295	1.69%	158.8562351	
H2-OE-A-H2-rep1_S35	13516638	9140710	3012411	12153121	89.91%	452749	237213	689962	5.10%	0.1449355182	0.1449355182
H2-OE-A-H2-rep2_S47	3902736	2465343	1120973	3586316	91.89%	78804	37718	116522	2.99%	0.8582070339	0.8582070339
H2-OE-M-H2-rep1_S36	22919056	16037311	5102317	21139628	92.24%	366899	176609	543508	2.37%	0.1839899321	0.1839899321
H2-OE-M-H2-rep2_S48	2478728	1702458	598187	2300645	92.82%	27287	13109	40396	1.63%	2.475492623	2.475492623
HSF2-neg-neg-Async-HSF2-1_S54	3916539	3036363	606294	3642657	93.01%	58991	12697	71688	1.83%	1.394933601	1.394933601
HSF2-neg-neg-Async-HSF2-3_S62	45024512	32185142	10559189	42744331	94.94%	669156	355314	1024470	2.28%	0.09761144787	0.09761144787
HSF2-neg-neg-Mit-HSF2-1_S56	23557588	16737106	4957337	21694443	92.09%	414187	142487	556674	2.36%	0.07963835207	0.07963835207
HSF2-neg-neg-Mit-HSF2-2_S57	7709890	5352229	1460232	6812461	88.36%	156777	56528	213305	2.77%	0.3688122641	0.3688122641

HA-HSF2-FL-Async-rep1_S33	19020855	12428577	5177435	17606012	92.56%	441852	185926	627778	3.30%	0.159291979	0.159291979
HA-HSF2-FL-Async-rep3_S37	12553352	8153395	3538790	11692185	93.14%	200832	88713	289545	2.31%	0.345369459	0.345369459
HA-HSF2-FL-Mit-rep2_S36	30508883	17013749	11829720	28843469	94.54%	458567	173911	632478	2.07%	0.1581082662	0.1581082662
HA-HSF2-FL-Mit-rep3_S38	15050976	9818865	4570997	14389862	95.61%	118428	54748	173176	1.15%	0.5774472213	0.5774472213
HA-HSF2-DBD-Async-rep1_S39	14170241	9029678	4186572	13216250	93.27%	244596	102340	346936	2.45%	0.2882376	0.2882376
HA-HSF2-DBD-Async-rep2_S41	34056472	23657843	9092546	32750389	96.16%	289391	126109	415500	1.22%	0.2406738869	0.2406738869
HA-HSF2-DBD-Mit-rep1_S40	18267207	11688632	5593821	17282453	94.61%	260786	101090	361876	1.98%	0.2763377511	0.2763377511
HA-HSF2-DBD-Mit-rep2_S42	6649479	4193013	2041982	6234995	93.77%	101795	39036	140831	2.12%	0.7100709361	0.7100709361
10-90-HSF2-1_S66	2402387	1467806	682111	2149917	89.49%	89365	37655	127020	5.29%	0.7872775941	0.7872775941
10-90-HSF2-2_S67	10780009	7627776	2635157	10262933	95.20%	129361	73234	202595	1.88%	0.4935955971	0.4935955971
5-95-HSF2-1_S69	29932425	21368655	7270917	28639572	95.68%	465737	224982	690719	2.31%	0.1447766747	0.1447766747
5-95-HSF2-3_S71	29983170	20508477	7929161	28437638	94.85%	395535	217350	612885	2.04%	0.1631627467	0.1631627467
B8-HA-Ctrl-rep-1_S92	443446	235667	166882	402549	90.78%	11628	13274	24902	6.15%	8.031483415	B8 ctrl had 20% S2 spike-in
B8-HA-Ctrl-rep-2_S93	357876	208853	119303	328156	91.70%	8835	7704	16539	5.10%	12.09262954	B8 ctrl had 20% S2 spike-in
